# Significant Differences in the Effects of Nitrogen Doping on Pristine Biochar and Graphene-like Biochar for the Adsorption of Tetracycline

**DOI:** 10.3390/molecules29010173

**Published:** 2023-12-27

**Authors:** Lingling Rong, Ligui Wu, Tiao Zhang, Cui Hu, Haihui Tang, Hongcheng Pan, Xiaoming Zou

**Affiliations:** 1 College of Environmental Science and Engineering, Guilin University of Technology, Guilin 541004, China; ronglingling1616@163.com; 2 School of Life Science, Jinggangshan University, 28 Xueyuan Road, Ji’an 343009, China; zhangtiao2020@163.com (T.Z.); hucui912@126.com (C.H.); tanghaihui_1995@163.com (H.T.); 3 College of Environmental Science and Engineering, Tongji University, Shanghai 200092, China; wuligui1229@126.com

**Keywords:** nitrogen doping, biochar, tetracycline antibiotics, adsorption mechanism, density functional theory

## Abstract

To improve the adsorption efficiency of pollutants by biochar, preparing graphene-like biochar (GBC) or nitrogen-doped biochar are two commonly used methods. However, the difference in the nitrogen doping (N-doping) effects upon the adsorption of pollutants by pristine biochar (PBC) and GBC, as well as the underlying mechanisms, are still unclear. Take the tetracycline (TC) as an example, the present study analyzed the characteristics of the adsorption of TCs on biochars (PBC, GBC, N-PBC, N-GBC), and significant differences in the effects of N-doping on the adsorption of TCs by PBC and GBC were consistently observed at different solution properties. Specifically, N-doping had varied effects on the adsorption performance of PBC, whereas it uniformly improved the adsorption performance of GBC. To interpret the phenomenon, the N-doping upon the adsorption was revealed by the QSAR model, which indicated that the pore filling (*V_M_*) and the interactions between TCs with biochars (*E_ad-v_*) were found to be the most important two factors. Furthermore, the density functional theory (DFT) results demonstrated that N-doping slightly affects biochar’s chemical reactivity. The van der Waals (vdWs) and electrostatic interactions are the main forces for TCs-biochars interactions. Moreover, N-doping mostly strengthened the electrostatic interactions of TCs-biochars, but the vdWs interactions of most samples remained largely unaffected. Overall, the revealed mechanism of N-doping on TCs adsorption by biochars will enhance our knowledge of antibiotic pollution remediation.

## 1. Introduction

The water environment is threatened by various pollutants due to rapid industrialization, such as heavy metals and organic pollutants, which is considered the main problem affecting the sustainable development of human beings [1]. Especially, the global use of antibiotics in both livestock and humans has increased significantly since the outbreak of COVID-19. This has led to a concerning level of antibiotic pollution in water bodies, which poses a serious threat to public health and the environment [2]. Tetracycline antibiotics (TCs) are widely used antimicrobials that inhibit bacterial protein synthesis by hindering the binding process between aminoacyl-tRNA and ribosomes [3]. These antibiotics possess broad-spectrum antimicrobial properties and have found extensive application in human therapy, animal disease control, as well as feed additives in livestock and aquaculture [4,5,6]. However, the pervasive misuse and overuse of TCs in recent decades have led to their steady discharge into various aquatic environments. Regrettably, TCs have a stable chemical structure that hinders their biodegradation, resulting in the presence of approximately 75% of parent compounds in groundwater, wastewater, and drinking water at ng·L^−1^ to μg·L^−1^ concentrations [7]. This is a cause for significant concern, as residual TCs have been observed to exhibit toxic effects on aquatic organisms, including bacteria, algae, and crustaceans. Moreover, their presence stimulates the proliferation of antibiotic resistance genes (ARGs) and disrupts the ecosystem balance of the original microbial community [8]. In some cases, this disruption has even led to the emergence of superbugs [9]. Consequently, it is imperative to address the removal of TCs from aquatic environments to ensure the continued health of these ecosystems [10].

Numerous treatment techniques have been employed to remove TCs from the polluted aquatic environment [11], including biological wastewater treatment plants (WWTPs), electrochemistry, membrane separation, advanced oxidation processes (AOPs), and adsorption [12]. However, conventional WWTPs often have insufficient removal efficiency for TCs due to their stable chemical structure, which is toxic to bacteria and resistant to biodegradation [13]. On the other hand, AOPs can achieve high removal efficiency for TCs due to the presence of highly reactive radicals [14]. Nevertheless, the practical application of AOPs is limited by their expensive operation, the formation of intermediate products, and the low concentration of TCs in real environments [15]. Adsorption, on the other hand, provides a practical solution to water contaminated with TCs due to its safety, low cost, and ease of operation. Recently, a variety of absorbents, including activated carbon [16], zeolite [17], biochars [18], and other porous materials, have been used. Biochar, in particular, is a carbon-rich material with a large specific surface area (SSA), rich functional groups, and a high cation exchange capacity. It can be produced from waste feedstock [19]. However, concerns persist regarding the removal of TCs and other antibiotics by biochars due to their excellent adsorption performance and economic benefits [20].

In general, commonly used biochar exhibits natural deficiencies, such as low hydrophilicity, insufficient surface active sites, and the generation of numerous tar-like substances and amorphous carbon during the pyrolysis process. These factors have been observed to decrease the SSA of biochar, and, consequently, result in a reduction in its adsorption efficiency for pollutants [21]. To overcome these limitations, various strategies have been proposed to develop novel modified biochars, which can be classified into chemical modification (e.g., acid modification, alkalinity modification, oxidizing agent modification, metal salts) and physical modification (e.g., steam and gas purging) approaches [22]. Among these approaches, alkali treatment has been proven to effectively increase the surface area, pore volume, and number of functional groups on the surface of biochar [23]. Consequently, alkali-prepared graphene-like biochar (GBC), a novel type of material, has recently gained attention as an adsorbent and support material [24]. Furthermore, the surface properties of biochar can be enhanced by incorporating heteroatoms, such as nitrogen (N), sulfur (S), and phosphorus (P), which have been demonstrated to improve biochar’s unique adsorption performance [25]. However, the specific effects of N-doping on the adsorption performance of GBC and pristine biochar (PBC) in the case of antibiotic adsorption have yet to be fully elucidated.

The adsorption process typically involves multiple stages, including solute transportation, diffusion across the liquid film, diffusion within the liquid, and interactions with active sites [18]. Various factors influence the adsorption performance, such as the structural characteristics of the adsorbate, the properties of the adsorbent surface, and the adsorption interface [26]. Specifically, previous studies showed that the π-π interaction and SSA of biochar are increased after N-doping, which improves the adsorption capacity for bisphenol A and phenol [27,28]. For the adsorption of TCs onto adsorbents, the π-π interaction is known to be crucial in the adsorption process of nitrogen-doped (N-doped) lignin-based carbon material [29]. However, the details of how N-doping affects the adsorption performance of GBC and PBC for TCs are yet to be fully understood. Understanding these mechanisms is crucial for the development of more effective adsorbents for TCs removal.

Density functional theory (DFT) is a potent method for quantitatively investigating the micro-mechanisms of adsorption between biochars and antibiotics [30]. Oxytetracycline (OTC), tetracycline (TC), and other tested TCs are frequently found in polluted aquatic environments and have been extensively studied to understand their adsorption behaviors [31,32]. Therefore, the objectives of this study are to (1) evaluate the influence of N-doping on PBC and GBC in terms of TC adsorption, and (2) elucidate the underlying mechanisms behind the differential impact of N-doping on TCs adsorption efficiency in PBC and GBC.

## 2. Results and Discussion

### 2.1. Characterization of the Tested Biochars

**Figure 1:**
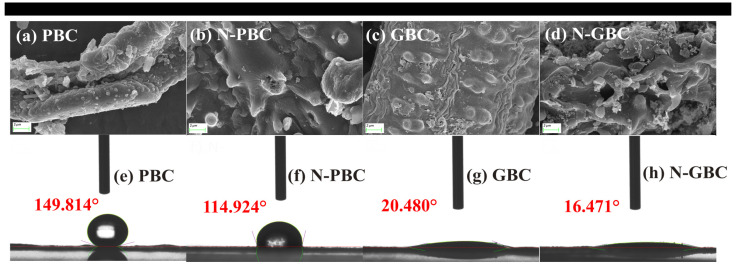
SEM images (5000-fold) (**a**–**d**) and contact angles (**e**–**h**) of tested biochars.

**Figure 2:**
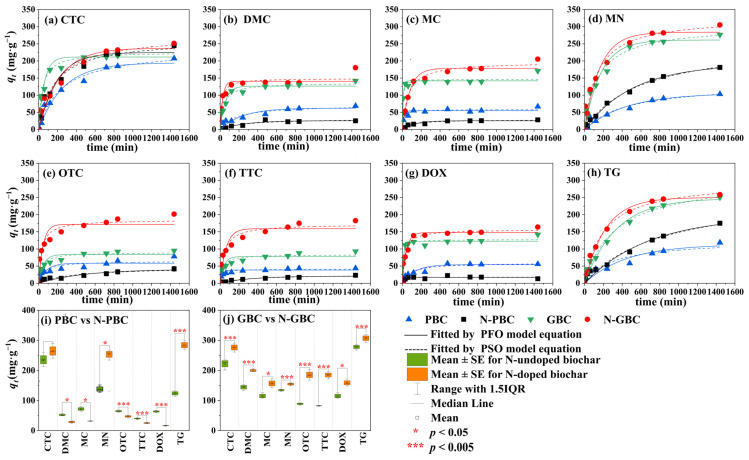
Fitted kinetic curves by the model of PFO and PSO of tested TCs adsorbed by N-doped and N-undoped biochars (**a**–**h**) and the statistical distribution of their TCs adsorption efficiency (*q_t_*) (**i**,**j**).

The basic physical and chemical properties of the four tested biochars were determined, including the surface morphology, atomic ratios, and point of zero charge (pH_PZC_) (Figure 1 and Table S1). The elemental composition of the tested biochars is listed in Table S1. As shown, the order of the molar ratios for O/C, (O+N)/C, and H/C was N-GBC > GBC > N-PBC > PBC, N-GBC > N-PBC > GBC > PBC and GBC > N-GBC > PBC > N-PBC, respectively. It can be concluded that (1) N-doped biochars possessed a higher content of N element (>4.50%) than those of N-undoped biochars (<0.50%), which was consistent with the SEM results of Figure 1; (2) a higher total percentage of O combined N was obtained in the N-doped biochars (>34.40%) than that of N-undoped biochars (<19.60%). Those results suggested that higher hydrophobicity (O/C) and polarity ((N+O)/C) and lower aromaticity (H/C) were obtained in N-doped biochars (N-PBC, N-GBC) [33]. Consequently, it is reasonable to speculate that the N-doping process may cause different effects on the TCs adsorption performance of PBC and GBC.

### 2.2. Adsorption Characteristics

#### 2.2.1. Adsorption Kinetics

The kinetics of adsorption are frequently employed to investigate the time required to attain adsorption equilibrium and to discern potential adsorption mechanisms [34]. As shown in Figure 2 and Table S2, it was observed that the PSO model (*R*^2^ > 0.90) provided a better fit for the adsorption data than the PFO model (*R*^2^ > 0.80), indicating that chemisorption is likely the primary adsorption process involved in the tested biochars [35]. Notably, the fast adsorption process of five TCs (DMC, DOC, OTC, TTC, and DOX) onto the tested biochars (PBC, N-PBC, GBC, and N-GBC) was achieved with almost 90% adsorption quantity within 100 min and near equilibrium level at 200 min (Figure 2a–h). However, adsorption equilibrium was not reached until roughly 800 min for the other three TCs (CTC, MN, and TG). Furthermore, according to the fitted results of the PSO model, the adsorption efficiency of TCs (*q_t_*) for PBC, N-PBC, GBC, and N-GBC ranged from 41.16 mg·g^−1^ to 235.51 mg·g^−1^, 17.80 mg·g^−1^ to 270.25 mg·g^−1^, 83.58 mg·g^−1^ to 308.09 mg·g^−1^, and 135.91 mg·g^−1^ to 327.20 mg·g^−1^, respectively. 

To further analyze the difference among samples, paired *t*-test analysis was performed on the above data (Figure 2i,j). The results showed that except for the groups of GBC/N-GBC for MN, OTC, and TTC, there is a statistically significant difference (*p* < 0.05) or very significant difference (*p* < 0.005) between samples with the corresponding N-doped samples, indicating the significant results of N-doping upon the adsorption of TCs by PBC and GBC. Specifically, it was determined that N-doping had differential effects on adsorption efficiency, depending on whether the biochar was a pristine group (PBC, N-PBC) or a graphene-like group (GBC, N-GBC). In the case of the pristine group, N-doping improved or reduced adsorption performance, as evidenced by the fact that the *q_t_* values of N-PBC increased from −69.988% to 116.096% compared with PBC. Conversely, N-doping consistently improved the adsorption performance for the graphene-like group, where the *q_t_* values of N-GBC increased from 12.90% to 106.23% compared to GBC.

#### 2.2.2. Adsorption Isotherm

**Figure 3:**
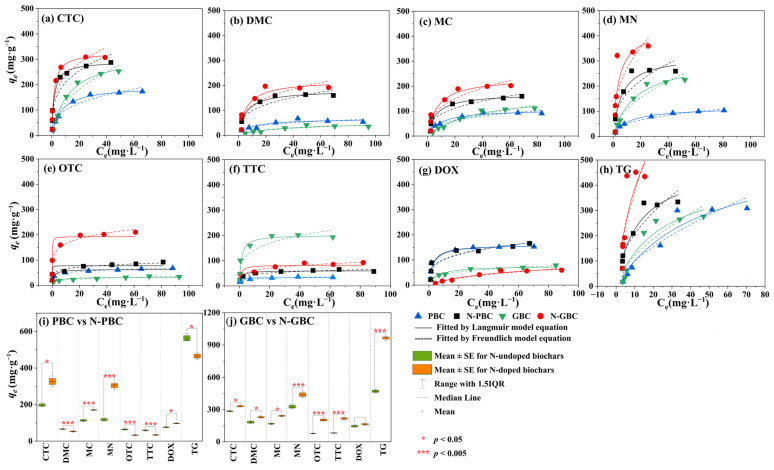
Fitted kinetic curves by the model of Langmuir and Freundlich of eight TCs.

Adsorption isotherm models are commonly utilized to characterize the interaction mechanisms between adsorbent and adsorbate, including the distribution of adsorbates in aqueous solutions [36]. In the present study, experimental data were fitted using both the Freundlich and Langmuir models, with the latter demonstrating superior accuracy in describing the adsorption of seven TCs (CTC, DOX, MC, TG, DMC, TTC, and MN) onto tested biochars (Figure 3). The fitting analysis revealed that the R^2^ values for the Langmuir model ranged from 0.630 to 0.999, while those for the Freundlich model ranged from 0.930 to 0.998, indicating that the Freundlich model exhibited a better fit for describing the adsorption of OTC on biochar. These results suggested that the adsorption process of TCs onto the tested biochars was primarily monolayer rather than multilayer adsorption [18]. Furthermore, significant differences were observed between the effects of N-doping on the adsorption performance of the pristine group (PBC, N-PBC) and graphene-like group (GBC, N-GBC). Based on the fitted results of the Langmuir model (Table S3), it was observed that the maximum adsorption capacity (*q*_m_) for PBC, N-PBC, GBC, and N-GBC varied from 60.260 mg·g^−1^ to 552.290 mg·g^−1^, 32.800 mg·g^−1^ to 464.810 mg·g^−1^, 77.860 mg·g^−1^ to 491.110 mg·g^−1^, and 160.840 mg·g^−1^ to 942.710 mg·g^−1^, respectively. When it comes to the pristine group (PBC, N-PBC), the effect of N-doping on the adsorption performance can be two-fold, as evidenced by the fact that the *q*_m_ values of N-PBC increased by −79.765% to 57.374% compared to PBC. On the other hand, for the graphene-like group (GBC, N-GBC), N-doping consistently improved the adsorption performance, as the *q*_m_ values of N-GBC increased by 7.108% to 141.934% compared to GBC. Additionally, it should be noted that the efficacy of N-doping for enhancing adsorption performance in graphene-like groups is influenced by the specific structural characteristics of the tested TCs. The outcome of the ANOVA revealed that there is a statistically significant distinction between the adsorption efficiency of TCs for GBC and N-GBC (*p* = 0.0472). Notably, N-GBC exhibits higher *q*_m_ values compared to GBC, with a pattern of increasing order as DMC < DOC < MN < CTC < MC < OTC < TTC < TG.

Overall, N-doping exhibits a remarkable strengthening effect on the adsorption performance of the graphene-like group (N-GBC, GBC). This phenomenon is closely correlated with the structural characteristics of the tested TCs. However, in the pristine group (PBC, N-PBC), the impact of N-doping on the adsorption efficiency may vary.

### 2.3. Influence of Solution Properties

To further verify the aforementioned conclusion, the impact of solution properties on the adsorption of TCs by the tested biochars was assessed under conditions of varying pH values (3–11), humic acid (HA) concentrations (0–30 mg·L^−1^), and salinity concentrations (0–10 mg·L^−1^). Results presented in Figure S1a,b indicated that the adsorption of TTC and OTC onto the tested biochars was significantly influenced by solution pH (*p* < 0.01). Changes in salinity can significantly impact the electrostatic interactions between chemicals and biochars, subsequently altering their adsorption features [37]. As shown in Figure S1c,d, our study found that the addition of Ca^2+^ had no significant effects on the adsorption of OTC-GBC (*p* > 0.05), but did have significant effects on other adsorption processes (*p* < 0.01 or *p* < 0.05). HA is a typical dissolved organic matter with π electrons, aromatic rings, and fatty acids. As presented in Figure S1e,f, the HA has significant effects on the adsorption of tested TCs (OTC, TTC) (*p* < 0.01). Overall, results of solution properties indicate that (1) the significant strengthening and complex effects of N-doping on the TCs adsorption performance can be obtained for PBC and GBC, respectively, and (2) the electrostatic interaction and π-π interaction are important mechanisms of TCs interacting with tested biochars, which can be changed by the N-doping process. Detailed information about the influence of solution properties upon adsorption features was listed in Supplementary Materials (SM, Text S1).

### 2.4. Mechanisms for the Effects of N-Doping upon the Adsorption Performance of TCs

According to the previous results [38], N elements in the N-doped biochar are mainly classified as chemical N and structural N, which significantly modify the surface in the form of N-containing functional groups and thereby change the SSA with a developed pore structure. In the present study, the molar ratios of H/C, O/C, and (O+N)/C for the tested biochars were different (Table S4). Consequently, it could be speculated that the structural differentiation of the tested biochars is the original driver resulting in the different characterization of TCs adsorption.

#### 2.4.1. Modification of Pore Structure

**Figure 4:**
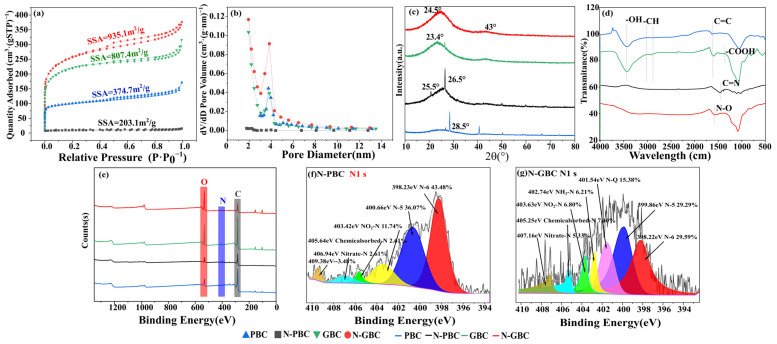
N_2_ adsorption and desorption isotherms (**a**), pore size distribution (**b**), XRD patterns (**c**), FTIR images (**d**), XPS diagrams (**e**) of tested biochars and XPS regional spectra of nitrogen in N-PBC and N-GBC (**f**,**g**).

The porous characteristics of tested biochars were analyzed by N_2_ adsorption and desorption isotherms, as shown in Figure 4a, which can be viewed as type I and IV adsorption isotherms. For all samples, the isotherms rapidly increased at P/P_0_ < 0.1, strongly demonstrating the presence of micropore structure in the biochars. The isotherms of GBC and N-GBC also indicated multilayer adsorption followed by capillary condensation, as proved by the obviously increasing adsorbing capacity at P/P_0_ > 0.9, indicating the presence of a mesopore structure in the GBC and N-GBC. It should be noted that the slightly increasing adsorbing capacity was obtained in N-PBC rather than in PBC. Furthermore, the pore size distribution of the tested biochars was proved to range from 2–5 nm (Figure 4b), confirming that the pores in the biochars were mainly mesopores. The positions of the most probable aperture size for PBC, N-PBC, GBC, and N-GBC were 3.122 nm, 3.611 nm, 4.931 nm, and 4.147 nm, respectively. The results indicate that the N-doping process slightly hinders the mesopore formation of N-PBC but aids the mesopore formation of N-GBC. A smaller pore diameter typically corresponds to a larger pore volume and SSA. As shown in Figure 4a, N-doping treatment significantly affected SSA and pore volume (*p* < 0.01). The SSA for PBC, N-PBC, GBC, and N-GBC was 374.67 m^2^·g^−1^, 203.11 m^2^·g^−1^, 807.41 m^2^·g^−1^, and 935.09 m^2^·g^−1^, respectively. The pore volumes for PBC, N-PBC, GBC, and N-GBC were 0.127 m^2^·g^−1^, 0.031 m^2^·g^−1^, 0.229 m^2^·g^−1^, and 0.294 m^2^·g^−1^, respectively. Obviously, those results consistently demonstrated that the N-doping process significantly decreased the adsorption sphere of PBC but significantly increased the adsorption sphere of N-GBC. Similar results were also obtained in the study of Diao et al. [39], which can be explained by the breaking down of carbon framework due to the gas produced by urea during pyrolysis [40].

#### 2.4.2. Modification of Surface Functional Groups

The short-range ordered structures of the tested biochars were revealed through XRD patterns, with the observed strong and sharp peaks indicating the presence of crystalline inorganic phases. Figure 4c shows that the broad peaks at 2θ = 23~29° were consistently observed and considered as amorphous structures of carbon [41,42], consistent with the SEM results in Figure 1. The peaks (2θ = 23~29°) of N-doped biochars (N-PBC, N-GBC) were flatter than those of PBC and GBC, indicating that N-doping improved the crystallinity of the carbon in these biochars [38]. Furthermore, FTIR spectra were determined to verify the main functional group of the tested biochars (Figure 4d). As shown, the N-undoped biochars exhibited similar FTIR spectra, with major bands occurring at peaks around 3436 cm^−1^ (–OH), 1514 cm^−1^ (–COOH), 1590 cm^−1^ (aromatic C=O and C=C), 1400 cm^−1^ and 2900 cm^−1^ (–CH), and 1050 cm^−1^ (–SiO_3_^2−^) [18]. Compared to the N-undoped group, the important peaks of N-PBC and N-GBC were shifted, strengthened, or weakened. Specifically, the vibration intensity of 3436 cm^−1^ (–OH) was weakened, and similar results were also obtained in the study of Li et al. [43], which can be mainly attributed to the replacement of O atoms by the N-doped structures (e.g., pyridinic-nitrogen, pyrrolic-nitrogen, and graphitic-nitrogen) [38]. The vibration intensity of around 1600 cm^−1^ (C=N) was strengthened, indicating the successful doping of N atoms in the carbon network structure [44]. The vibration intensity of around 1514 cm^−1^ (–COOH) was also modified, suggesting that the –COOH group was affected by the N-doping process [45].

To analyze the functional groups of the tested biochars on a quantitative level, the XPS spectra were further determined because XPS measurement is widely used to analyze the binding relationship between elements [46]. Figure 4e illustrates the dominance of electron energy spectra peaks associated with C1s and O1s primarily on the surfaces of PBC and GBC, delineating carbon (C) and oxygen (O) as the predominant constituents of rice husk. This observation aligns consistently with elemental analysis results. However, subsequent biochar modification employing C_2_H_4_N_4_ induced notable alterations in elemental composition. Remarkably, N contents in N-PBC and N-GBC exhibited substantial escalation, with increments of 9.08% and 45.28%, respectively, in comparison to their precursor PBC. Zhou et al. utilized N dopants—such as amides, triethanolamine, and ethylenediamine—to augment biochar derived from corn stover, achieving heightened N content and bolstering CO_2_ adsorption capabilities [47]. Additionally, scrutiny of the C1s, O1s, and N1s spectra (Figure 4e,f and Figure S2) revealed the deconstruction of combined C1s and O1s relationships into four distinct C functional groups and three O functional groups. Notably, N1s spectroscopy predominantly identified three types of N functional groups: pyridinic-nitrogen (398.23 eV, 398.22 eV), pyrrolic-nitrogen (400.66 eV, 399.86 eV), and graphitic-nitrogen (401.54 eV). Previous studies elucidate that within N-doped biochar, pyridinic-nitrogen emerges as the principal active N species, pivotal in initiating the activation of peroxymonosulfate and facilitating the degradation of ciprofloxacin processes [48]. Consequently, this infers that within this study, the emergence of heterocyclic nitrogen configurations (e.g., pyrrolic, pyridinic, and graphitic nitrogen) subsequent to the further modification of GBC into N-GBC potentially underlies its augmented adsorption capabilities, given the higher reactivity exhibited by these heterocyclic nitrogen species [49].

#### 2.4.3. Mechanistic Model Developments and Model Explanation

Previous studies demonstrated that biochar-driven adsorption of antibiotics is predominantly influenced by pore filling, ion exchange, hydrogen bonding, and π-π energy decomposition analyses (EDA) interactions [50]. As is well known, the adsorption process of TCs in solution involves several steps, including dissolution, migration or diffusion, and interaction with biochars [51]. To better understand the roles of the above steps, the log*K_ow_* values of the tested TCs were calculated, as log*K_ow_* has proven to be an important factor in determining the uptake, distribution, and elimination of antibiotics [52]. To describe the effects of pore filling, the parameters of SSA and molecular volume (*V_M_*) were used because (1) the SSA values were derived from the curve of the pore-filling in the adsorption (Figure 4a), and (2) pore filling is tightly linked to the molecular volume of the tested chemicals [53]. To measure the interactions with the biochars, the valid adsorption energies (*E_ad-v_*) were developed because, (1) the adsorption energy (*E*_ad_) rationally describes the strength between such interactions as ion exchange, H-bond and π-π EDA interaction [18,54] and (2) *E_ad-v_* exactly describes the total adsorption interactions of TCs on the biochar [51], according to the proportion of different biochar models (i.e., pyridinic-nitrogen, pyrrolic-nitrogen and graphitic-nitrogen). Therefore, the adsorption efficiency of TCs (*q_e_*) and three parameters relating to adsorption mechanisms (log*K_ow_*, log(*SSA*), *V_M_*, *E_ad-v_*; Tables S4 and S5) are used as independent and dependent, respectively. The mechanistic models were developed in Equations (1) and (2) for pristine biochars (PBC, N-PBC) and graphene-like biochars (GBC and N-GBC), respectively.

(1) $$q_{e}=-1912.160-0.648\times E_{ad-v}+3.869\times V_{M}-12.571\times {logK}_{ow}$$

*n* = 14, F = 10.715, *R*^2^ = 0.763, *Q*^2^_(cum)_ = 0.618, *SE* = 130.159, *p* = 0.002; *n*_(Ext)_ = 2, *Q*^2^ _(ext)_ = 0.503, *SE*_(Ext)_ = 171.178

(2) $$q_{e}=-2611.753-0.381\times E_{ad-v}+8.773\times V_{M}+79.3156\times {logK}_{ow}$$

*n* = 14, F = 17.078, *R*^2^ = 0.837, *Q*^2^_(cum)_ = 0.516, *SE* = 151.417, *p* = 0.000; *n*_(Ext)_ = 2, *Q*^2^ _(ext)_ = 0.518, *SE*_(Ext)_ = 145.928

**Figure 5:**
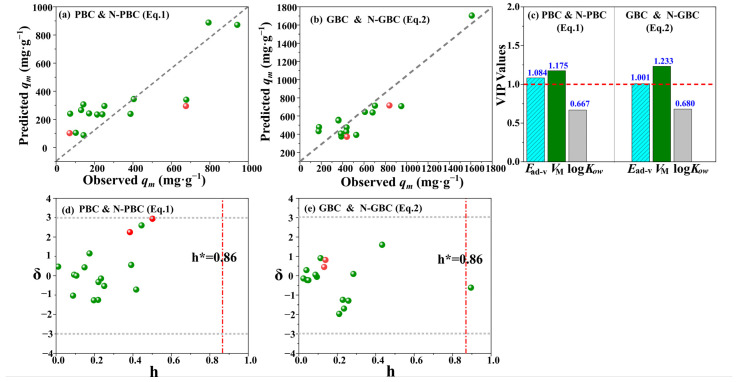
The correlation of predicted and observed adsorption capacity *q*_m_ (**a**,**b**), VIP values of variables (**c**), and Williams plots (**d**,**e**) for the developed QSAR model. The red and grey dotted lines present the threshold of *h* and δ.

Based on the analysis of various parameters and Williams plots, it can be concluded that the developed models are of good quality (Figure 5). This is attributed to the fact that the *R*^2^ and *Q*^2^ values exceeded the 0.50 threshold and there were no outliers observed for the response, as evidenced by the low standardized residuals (*δ*) of the tests (<3) (Figure 5d,e). Moreover, the *h*_i_ values of the tested data consistently exhibited lower values than the corresponding h* values, indicating the absence of influential predicted values in the mode space [55]. The h* is the warning value of *h_i_*. Hence, it can be stated that the developed Equations (1) and (2) models can effectively elucidate the adsorption mechanism of TCs by tested biochars. Furthermore, the variable importance in the projection (VIP) values (Figure 5c) in Equations (1) and (2) is uniformly *V_M_* > *E_ad-v_* > log*K_ow_*, the VIP values of *V_M_* and *E_ad-v_* are consistently above 1.0, suggesting the fact that the effects of pore filling (*V_M_*) and the interactions between TCs with the tested biochars, tend to be important processes for the adsorption characteristics of TCs by the tested biochars [56].

### 2.5. The Micro-Mechanisms of N-Doping Effects upon PBC and GBC for the Adsorption of TCs

#### 2.5.1. Effects of N-Doping upon the Chemical Reactivity of Tested Biochars

**Figure 6:**
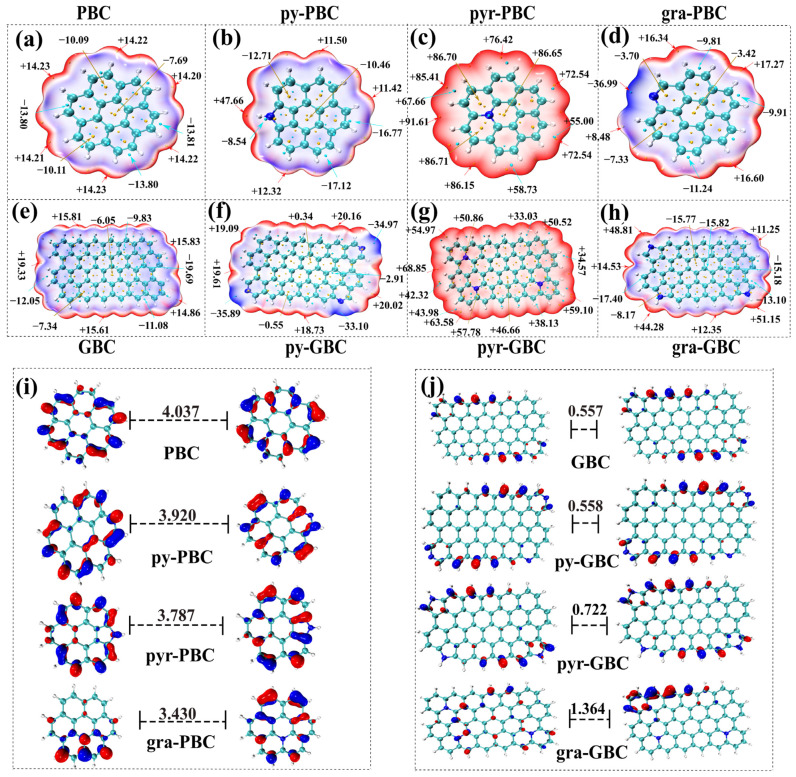
The electrostatic potential (**a**–**h**) and 3D isosurface plots of the HOMO and LUMO orbital (**i**,**j**) of tested biochars.

The significance of electrostatic interaction and π-π EDA interaction were highlighted as mechanisms for TCs interacting with the biochars in Figure 3. Therefore, The ESP of the studied absorbents was calculated to give an overview of the adsorption phenomenon at the point of charge distribution. As shown in Figure 6a–h, the tested biochars were colored blue, white, and red. According to the color transition description [57], the red, white and blue colors represent, respectively, a positive electrostatic potential, a vanished ESP value region, and a negative electrostatic potential. Thus, the red and blue colors indicate the region of an electron-deficient or an electron-rich, respectively. As shown in Figure 6, N-doping exhibits remarkable effects on the electron-deficient region and the electron-rich region. The values on the vdWs surface for PBC, py-PBC, pyr-PBC, and gra-PBC are ranging from −13.81 to 14.23 kcal·mol^−1^, −17.12 to 47.66 kcal·mol^−1^, 55.00 to 91.61 kcal·mol^−1^, −36.99 to 17.27 kcal·mol^−1^, respectively. Whereas the values on the vdWs surface for GBC, py-GBC, pyr-GBC, gra-GBC are ranging from −19.69 to 14.23 kcal·mol^−1^, −35.89 to 19.61 kcal·mol^−1^, 34.57 to 68.85 kcal·mol^−1^, −17.40 to 51.15 kcal·mol^−1^, respectively. Therefore, it can be concluded that N-doping strengthens the positive electrostatic potential of all tested biochars while improving the negative electrostatic potential of partial biochars (except for pyr-PBC and pyr-GBC). Furthermore, as shown in Figure 6, the maximum values are mostly located in the H atoms around the N containing groups, which should have a strong ability to electrostatically attract negatively charged atoms and act as a hydrogen bonding donor group (i.e., Figure 6b,c,g,h). Also, it can be observed that if the N-containing groups have no H atoms, the minimum values were also detected around the N atoms and should be the most favorable nucleophilic sites when interacting with tested TCs (i.e., Figure 6d,f). It should be noted that the above results are mainly caused by the fact that, (1) the incorporation of the N atom in the lattice induces a change in the hybridization of carbon atoms (sp3 → sp2), which significantly changes the electronic distribution of tested biochars [58], and (2) the N atom poses strong electronegativity due to its lone pair electrons [59].

Furthermore, in the field of chemistry, the frontier electron density commonly uses molecular orbitals to describe the reactive characteristics of chemicals in π-electron systems. LUMO accepts electrons and HOMO is associated with the electron donor. A molecule with a small HOMO-LUMO gap (*E*_h-l_) typically indicates its high global chemical reactivity [60]. As shown in Figure 6i,j, the *E*_h-l_ values of pristine biochars and graphene-like biochars ranged from 3.430–4.037 eV and 0.557–1.364 eV, respectively. *E*_h-l_ value of biochar is higher than that of other N-doped biochars (py-PBC, pyr-PBC, gra-PBC), whereas *E*_h-l_ value of GBC is lower than that of other N-doped biochars (py-GBC, pyr-GBC, gra-GBC). It is obvious that the results suggested that (1) higher chemical reactivity can be obtained in graphene-like biochars compared to pristine biochars, and (2) the chemical reactivity of tested biochars was consistently affected by N-doping. In detail, the N-doping process slightly increased the chemical reactivity of pristine biochars whereas weakly decreased the chemical reactivity of graphene-like biochars.

#### 2.5.2. Effects of N-Doping upon the Interactions of Biochars with TCs

##### Equilibrium Configurations and Adsorption Sites

**Figure 7:**
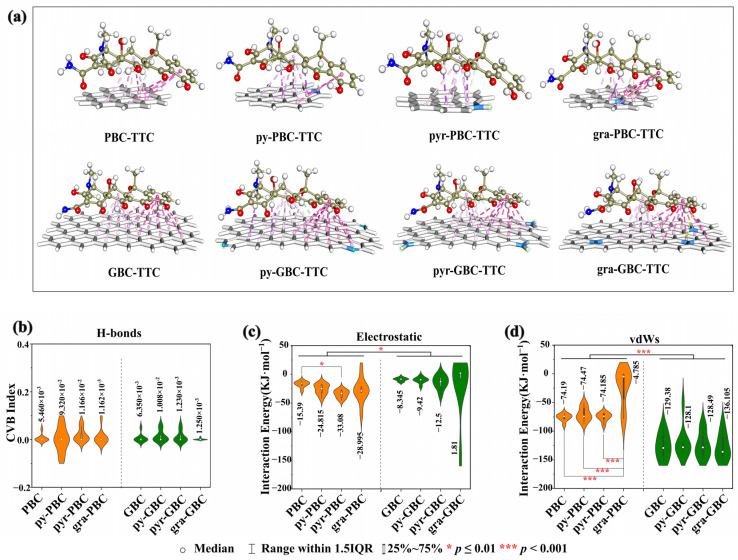
The lowest energy configurations of TTC-biochars (**a**), CVB index of H-bond (**b**), interaction energy of electrostatic (**c**) and vdWs (**d**) for TCs-biochars.

To further investigate the impact of N-doping on the interactions between biochars and TCs, we employed graphene structures consisting of 7 and 24 aromatic rings as models for PBC and GBC, respectively [18]. To further illustrate this, we can refer to Figure 7a (the example of TTC), which demonstrates the following: (1) the lowest energy configuration uniformly exhibits a plane-shaped geometry when TTC is absorbed onto the biochars; (2) a high level of consistency is observed in the adsorption sites of TTC on the biochar surface. Specifically, the D ring, B ring, and the –C=O group in the C ring serve as common sites for TTC binding with biochars; (3) the impact of N-doping on the adsorption sites of TCs with biochars is also evident in certain samples. For instance, only the B ring and the –C=O group in the C ring showed interactions with biochar in the adsorption of pyr-PBC-TTC, while the –OH group of the C ring interacted with the N group of biochar during the adsorption of py-PBC-TTC. It is worth noting that a similar phenomenon can be observed in other TCs-biochar adsorptions (Figure S3) and other published works [61].

##### Weak Interaction Forces

Based on the acquired equilibrium configurations, we conducted DS and IGMH analyses to elucidate the intricate interactions between the analyzed TCs and biochars (Figure 7, Figure S3, Table S6). The DS results consistently revealed that the equilibrium configurations of TCs-biochars exhibited a small amount of hydrogen bonding (H-bond) and a large number of π-bonds. Specifically, the H-bonds were predominantly found in the interactions between the –C=O group in the C ring of TTC and biochar, while the π-bonds were primarily identified in the interactions between the D and B rings of TTC and biochar. For example, PBC-TTC and py-GBC-TTC formed one and three H-bonds, respectively (Figure 7a). Furthermore, the IGMH analysis results (Figure S4) also confirmed this conclusion. The spike with a blue color (H-bond) was hardly observed in the interactions of TCs-biochars, whereas the spikes with green (vdWs interaction) and red (electrostatic interaction) colors were present in all interactions of TCs-biochars. Therefore, the weak forces for the adsorption of TCs-biochars consisted mainly of vdWs interactions and electrostatic interactions.

To quantitatively analyze the characteristics of H-bond, vdWs, and electrostatic interactions in relation to adsorption, the CVB index was calculated based on topological characterization, and the vdWs interaction energies (*E*_vdWs_) and the strength of electrostatic interactions (*E*_es_) were obtained using EDA-FF [57,62]. As illustrated in Figure 7b, the average CVB values for the pristine group (PBC, N-PBC) and the graphene-like group (GBC, N-GBC) ranged from 5.460 × 10^−3^ to 1.166 × 10^−2^ and 1.250 × 10^−3^ to 1.230 × 10^−2^, respectively. However, the CVB values in many samples were calculated to be zero. Generally, a smaller CVB index corresponds to a stronger H-bond interaction, and H-bond interactions with CVB values below zero are typically stronger [51]. Therefore, the CVB results indicated the weak H-bonding interactions in the adsorption of TCs-biochars. Regarding the electrostatic interaction (Figure 7c), the average *E*_es_ values for the pristine group (PBC, N-PBC) and the graphene-like group (GBC, N-GBC) ranged from −15.390 to −33.081 KJ·mol^−1^ and 1.810 to −12.510 KJ·mol^−1^, respectively. This suggests that (1) the electrostatic interactions of the graphene-like group are consistently and significantly weaker than those of the pristine group (*p* < 0.05), and (2) the N-doping process typically strengthens the electrostatic interactions of TCs-biochars, with the exception of gra-GBC-TCs. For example, the average *E*_es_ value of PBC-TCs is −15.390 KJ·mol^−1^, while the average *E*_es_ values of pyr-PBC-TCs and gra-PBC-TCs are −33.081 KJ·mol^−1^ and −28.995 KJ·mol^−1^, respectively. As for the vdWs interaction (Figure 7d) in the pristine group (PBC, N-PBC), there is no significant difference between PBC with py-PBC and pyr-PBC, whereas the average *E*_vdWs_ value of gra-PBC-TCs is increased to −4.785 KJ·mol^−1^. Interestingly, when it comes to the *E*_vdWs_ in the graphene-like group (GBC, N-GBC), there is no significant difference between GBC with py-GBC and pyr-GBC. However, the average *E*_vdWs_ value of gra-GBC-TCs decreases to −136.105 KJ·mol^−1^, suggesting that in comparison to N-undoped samples, the vdWs interaction of gra-GBC is significantly strengthened, while that of gra-PBC is significantly weakened by the N-doping process (*p* < 0.05).

##### Key Quantum Chemical Descriptors of TCs for Adsorption Energies

Based on the results of the adsorption isotherm and kinetics, we have observed that N-doping has a significant impact on the adsorption performance, which is closely related to the structural characteristics of the tested TCs (Figure 2, Figure 3 and Figure 4). In general, quantum chemical descriptors are commonly employed to quantitatively describe the structural features of TCs and unveil their mechanism of action [63,64]. Previous studies have also indicated that the quantum chemical descriptors of antibiotics indirectly influence their adsorption process [18]. Therefore, redundancy analysis (RDA) was conducted to unveil the relationships between quantum chemical descriptors and *E*_CVB_, *E*_vdWs_, and *E*_es_ (Figure S5). Specifically, in the pristine group (PBC, N-PBC), *E*_vdWs_ was principally affected by *ATSC5p* and *MATS5p*, *q*^H+^ and *q*^−^, *G*, *MAXDP2* in the interactions of PBC-TCs, py-PBC-TCs, pyr-PBC-TCs and gra-PBC-TCs, respectively. The major influencing factors for *E*_es_ were *q*^−^, *E*_lumo_ and *GATS6i*, *MATS4e*, *MinHBint* in the interactions of PBC-TCs, py-PBC-TCs, pyr-PBC-TCs and gra-PBC-TCs, respectively. On the other hand, in the graphene-like group (GBC, N-GBC), the principal influencing parameters for *E*_vdWs_ were *MATS5p*, *q*^−^ and *q*^H+^, *q*^−^ and *MDEC-33*, *q*^−^ and *q*^H+^ in the interactions of GBC-TCs, py-GBC-TCs, pyr-GBC-TCs and gra-GBC-TCs, respectively. The *E_es_* were primarily affected by the parameters of *E*, *SpMin1_B*, *μ*, *GATS5p* and *μ* in the interactions of GBC-TCs, py-GBC-TCs, pyr-GBC-TCs and gra-GBC-TCs, respectively. Therefore, we can conclude that: (1) there is a significant difference in the structural characteristics of the tested TCs between the N-doped biochars (N-PBC, N-GBC) and the N-undoped biochars (PBC, GBC) (*p* < 0.01), and (2) there is also a significant difference in the structural characteristics of the tested TCs that are affected by N-doping in the adsorption process between the pristine group biochars (PBC, N-PBC) and the graphene-like group biochars (GBC, N-GBC) (*p* < 0.01).

### 2.6. Application

Based on the N-undoped biochars (PBC, GBC) and N-doped biochars (py-PBC, pyr-PBC, gra-PBC, py-GBC, pyr-GBC, gra-GBC), the adsorption results (Figure 2 and Figure 3) proved that N-doping exhibits a remarkable strengthening effect on the adsorption performance in graphene-like group (N-GBC, GBC), and N-doping improves or reduces adsorption performance in pristine groups. Previous similar results showed that N-doping biochars increased the adsorption efficiency of heavy metals (Cu^2+^ and Zn^2+^) [65], dye [66], atrazine [59] and sulfonamide [24] on biochars. However, the previous works mainly focused on studying the adsorption kinetics and isotherm to reveal the mechanism. The current study not only examines the adsorption kinetics and isotherm but also incorporates QSAR models, molecular simulation results, and energy decomposition analysis. By doing so, the study provides a comprehensive understanding of the underlying mechanisms behind the substantial variations in the adsorption effects of N-doped pristine biochar and graphene-like biochar on TCs at a molecular level. This multi-faceted approach allows for a deeper interpretation of the observed differences and sheds light on the intricate molecular interactions involved in the adsorption process. As far as we know, this is the first report to comprehensively reveal the different N-doping effects upon the adsorption of antibiotic by pristine biochar and graphene-like biochar. Therefore, the present study undoubtedly provides more accurate theoretical guidance for further improving the adsorption efficiency of N-doped biochar.

## 3. Materials and Methods

### 3.1. Materials and Tested Biochars

In this study, eight TCs with a purity of over 99%, namely oxytetracycline (OTC, CAS: 79-57-2), tetracycline (TTC, CAS: 60-54-8), chlortetracycline (CTC, CAS: 57-62-5), minocycline (MN, CAS: 10118-90-8), tigecycline (TG, CAS: 220620-09-7), methacycline (MC, CAS: 914-00-1), doxycycline (DOX, CAS: 564-25-0), demeclocycline (DMC, CAS: 64-73-3) were purchased from Aladdin (https://www.aladdin-e.com/, accessed on 10 April 2021) and were used without any further purification. Table S4 lists the physicochemical properties and the quantum descriptors of the analyzed TCs.

The utilization of rice straw biomass material was favored due to its high adsorption effectiveness for TCs [67]. To prepare PBC, the rice straw was cleaned, dried, and pulverized. The pulverized powder was then placed into a tube furnace and heated in an N_2_ environment. The N-doping PBC (N-PBC) was synthesized through a direct pyrolysis process. To synthesize GBC, a comprehensive process with K_2_CO_3_ (CAS: 584-08-7, a purity of over 99%, Aladdin) was implemented. The process for generating N-doped GBC (N-GBC) was carried out using GBC and following a similar preparation method as that of N-PBC. Detailed information about the preparation of tested biochars was listed in Supplementary Materials (Text S2).

### 3.2. Characterization of Biochar and TCs Measurements

To elucidate the structural characteristics of the prepared biochars, a variety of analytical techniques were employed to assess their surface morphology, elemental composition, and functional groups. Specifically, the surface morphology was observed using scanning electron microscopy (SEM, Zeiss, Oberkochen, Germany), while the degree of crystallinity was determined through X-ray diffraction (XRD, Bruker, Bremen, Germany). Moreover, the contact angle was measured using a dynamic contact angle meter (Dataphysics DCAT11, Filderstadt, Germany), and the SSA and pore volume were analyzed using a surface area and porosity analyzer (Brunauer-Emmett-Teller (BET), Micromeritics, Norcross, GA, USA). The concentrations of carbon, hydrogen, oxygen, nitrogen, and sulfur were determined using an Element Analyzer (FLASH 2000, Thermo Fischer Scientific, Waltham, MA, USA). Fourier transform infrared spectrometry (FT-IR, Bruker, Bremen, Germany) was employed to investigate the functional groups, while X-ray photoelectron spectroscopy (XPS, PHI 5000, Kanagawa, Japan) was used to analyze changes in carbon, oxygen, and nitrogen. The detailed information about determining of point of zero charge and contact angle was listed in Supplementary Materials (Texts S3 and S4).

The concentration of TCs was assessed using an HPLC system (Shimadzu, LC-20AD, Kyoto, Japan) coupled with a C_18_ reverse-phase column. Detailed information about the measurement of tested TCs and the standard curves were listed in Supplementary Materials (Text S5 and Table S6).

### 3.3. Batch Adsorption Experiments

The adsorption of TCs onto biochars was conducted using a batch-type adsorption system under dark conditions. Specifically, a mixture containing 50 mL of TC solution and 10 mg of biochar was added to a 100 mL conical flask and agitated at 180 rpm and 25 °C for 48 h. Afterward, the resulting samples were filtered through a 0.22 μm filter, and the concentrations of TCs in the supernatant were analyzed using HPLC. Control groups were also included in the study to eliminate any deviations caused by the hydrolysis of TCs [68]. The effective adsorption data for all samples were determined by calculating the difference between the samples with and without biochars using Equation (3).

(3) $${\;\;\;C}_{i}^{r}=C_{i}^{C}-C_{i}^{S}$$

where $C_{i}^{r}$, $C_{i}^{C}$ and $C_{i}^{S}$ (mg·L^−1^) are the real adsorption concentrations of TCs in the sample solution at *i* time, the determined concentrations of TCs in the control solution at *i* time, and the determined concentrations of TCs in the sample solution at *i* time, respectively.

Based on the obtained results of $C_{i}^{r}$, the adsorption capacity (*q_t_* (mg·g^−1^)) and removal percentage (*P_e_*) were calculated by Equations (4) and (5).

(4) $$q_{t}=\frac{(C_{0}^{r}-C_{e}^{r})\times V}{m}$$

(5) $$P_{e}=\frac{\left(C_{0}^{r}-C_{e}^{r}\right)}{C_{0}^{r}}\times 100\%$$

where $C_{0}^{r}$ (mg·L^−1^) and $C_{e}^{r}$ (mg·L^−1^) are the initial and equilibrium concentrations of residual TCs in the solution, respectively. *m* (g) denotes the tested biochar weight, and *V* (L) denotes the volume of the TC solution.

To further characterize the adsorption features, the Langmuir model equation and the Freundlich model equation were employed to fit the adsorption isothermal data and evaluate the adsorption capacity of TCs on biochars. In addition, an investigation into the adsorption kinetics of biochars in relation to TCs was undertaken and the obtained adsorption kinetics data were fitted by the pseudo-first-order (PFO) model and the pseudo-second-order (PSO) model. Moreover, the influence of solution characteristics (pH values (3 to 11), HA concentrations (0 to 30 mg·L^−1^), and salinity concentrations (0 to 10 mg·L^−1^)) on the adsorption process was investigated. Detailed information about investigating adsorption features was listed in Supplementary Materials (Text S6). It is worth noting that the adsorption experiments in the present study were consistently conducted in triplicate, and control groups were established to evaluate the impact of the adsorbent’s absence.

### 3.4. Adsorption Simulation and Micro-Mechanisms

#### 3.4.1. Adsorption Simulation

To investigate the adsorption interfacial interactions between TCs and biochars, we employed graphene structures as models. The models comprised seven aromatic rings and 24 aromatic rings, representing the PBC model and the GBC model, respectively [18,69,70]. For N-doped biochars, we assigned specific names to distinguish them based on the modified groups of pyridinic-nitrogen, pyrrolic-nitrogen, and graphitic-nitrogen, namely py-PBC, pyr-PBC, and gra-PBC, respectively. Similarly, for the GBC models with pyridinic-nitrogen, pyrrolic-nitrogen, and graphitic-nitrogen, we named them py-GBC, pyr-GBC, and gra-GBC, respectively.

To determine the optimal low-energy configuration for TCs adsorption onto biochars, we employed the DMol3 program package within Materials Studio 2020 to conduct adsorption calculations using the models we have developed. As outlined in our previous investigation [18], the adsorption simulation specifics were as follows: The electronic basis set utilized was dual numerical polarization (DNP 4.4), the atomic basis function was an atom-centered grid, the self-consistent field convergence value was set at 1.0 × 10^−6^, and the core treatment was DFT semi-core pseudopots. After optimizing the biochars and TCs models, we employed the Metropolis Monte Carlo method to calculate absorption within the framework, utilizing 10^5^ Monte Carlo steps and 10 annealing cycles under the COMPASS II force field of the “adsorption locator calculation” program [68].

#### 3.4.2. Adsorption Micro-Mechanisms

To investigate the micro-mechanisms of adsorption, various parameters were studied, such as the adsorption energy (*E*_ad_), the valid adsorption energy (*E_ad-v_*), the equilibrium configurations of adsorption and their corresponding interaction features (such as bond angle, bond distance, and interaction types), the strength of interactions (including the vdWs interaction energies (*E*_vdWs_), the electrostatic interactions energies (*E*_es_), the core valence bifurcation (CVB) index for hydrogen bonding (H-bond) energy [54], and the electrostatic potential (ESP) [71]. Detailed information about obtaining the parameters of adsorption micro-mechanisms was listed in Supplementary Materials (Text S7).

### 3.5. Mechanistic QSAR Model Development, Validations, and Statistical Analyses

The adsorption features were analyzed through the development of quantitative structure activity relationship (QSAR) models. These models were created using partial least squares regression and evaluated based on metrics such as the determination coefficient (*R*^2^), the Fisher criterion (*F*), the standard error of estimate (*SE*), and the *p*-value test (*p*). In addition, cross-validation (*Q*^2^) and a Williams plot (standardized residuals (*δ*) versus leverage values (*h*_i_)) were used to verify the predictive ability and applicability of the model, respectively [55].

The statistical analysis was performed using SPSS 26.0 software (SPSS Inc., Chicago, IL, USA). A one-way analysis of variance and paired *t*-test were employed to compare the differences in samples, and a *p*-value lower than 0.05 was considered statistically significant.

## 4. Conclusions

In the adsorption of TCs onto tested biochars, N-doping may improve or reduce the adsorption performance of PBC, whereas consistently improves the adsorption performance of GBC. The above N-doping effects can obtain the same obtained in different solution properties. Furthermore, the N-doping process significantly modified the surface functional groups of biochars, decreased the adsorption sphere of PBC, and significantly increased the adsorption sphere of GBC. Moreover, the pore filling and the interactions between TCs and biochars were key factors in the effects of N-doping (VIP values > 1.0). Otherwise, electrostatic interactions and vdWs interactions were the primary weak forces, with the former being strengthened and the latter unaffected by the N-doping process. In conclusion, the proven mechanism for the different N-doping effects of TCs adsorption by biochars will enhance our understanding of the critical role of biochar in TCs pollution remediation.

Nevertheless, multiple contaminants often coexist in aquatic environments, necessitating a broader approach to water treatment. Although restricting research to a single pollutant provides valuable insights into its adsorption mechanisms, it would fail to address the complex challenges associated with water treatment comprehensively. Our study focused on the adsorption treatment of a specific pollutant (TCs), future research should prioritize the development of adsorbent materials capable of effectively removing multiple pollutants. To tackle this issue, future advancements should aim to synthesize or modify adsorption media to enhance their adsorption capacity and selectivity for a broader range of pollutants. Additionally, integrating advanced separation and treatment technologies, such as membrane filtration and adsorption resins, can facilitate the efficient removal and recovery of various pollutants. Interdisciplinary collaboration in research plays a crucial role in driving progress in the field of multi-pollutant adsorption treatment.

## Data Availability

Data will be made available on request.

## Supplementary Materials

The following supporting information can be downloaded at: https://www.mdpi.com/article/10.3390/molecules29010173/s1, Figure S1: The influence of environmental factors (pH, Ca^2+^, HA) on TTC and OTC adsorption efficiency (*q_e_*) by tested biochars (a–f) and the statistical distribution of their adsorption efficiency (g); Figure S2: C1s, O1s peaks of tested biochars by XPS regional spectra; Figure S3: Equilibrium configurations of tested TCs (except TTC) adsorbed by PBC and GBC (a), adsorbed by py-PBC and py-GBC (b), adsorbed by pyr-PBC and pyr-GBC (c), adsorbed by gra-PBC and gra-GBC (d); Figure S4: The scatterplots of reduced density gradients versus sign(λ2)ρ for the equilibrium configurations of TTC-biochars; Figure S5: The redundancy analysis (RDA) of quantum chemical descriptors and E_CVB_, E_vdWs_, and E_es_; Table S1: Characteristic of the tested biochars; Table S2: The fitted parameters of PFO model and PSO model; Table S3: The fitted parameters for Langmuir model and Freundlich model; Table S4: The physicochemical properties and the quantum descriptors of tested TCs; Table S5: The valid adsorption energies of TCs of TCs with tested biochars; Table S6: The interaction types, distances and angels of TCs-biochars; Table S7: The standard curves and recovery rates of tested TCs; Text S1: Adsorption features; Text S2: Preparation of tested biochars; Text S3: Determination of point of zero charge; Text S4: Determination of contact angle; Text S5: TCs measurements; Text S6: Adsorption micro-mechanisms; Text S7: Effects of environmental conditions. References are cited in [18,37,57,72,73,74].

## List of Abbreviations and Definitions

Graphene like biochar—GBC, pristine biochar—PBC, nitrogen doped GBC—N-GBC, nitrogen doped PBC—N-PBC, tetracycline antibiotics—TCs, valid adsorption energies—*E_ad-v_*, quantitative structure activity relationship—QSAR, octanol-water partition coefficients—*K_ow_*, specific surface area—SSA, molecular volume—*V_M_*, the core valence bifurcation index—CVB, hydrogen bonding—H-bond, van der Waals—vdWs, density functional theory—DFT, humic acid—HA, pseudo-first-order—PFO, pseudo-second-order—PSO, redundancy analysis—RDA, electrostatic potential—ESP, energy decomposition analyses—EDA.
