# Supplementary material for: Significant Differences in the Effects of Nitrogen Doping on Pristine Biochar and Graphene-like Biochar for the Adsorption of Tetracycline
Source: Molecules. 2023 Dec 27;29(1):173. doi: 10.3390/molecules29010173 (PMC10779899; doi:10.3390/molecules29010173)
Supplement: Supplementary file 1 [file molecules-29-00173-s001.zip › molecules-2773374-supplementary.pdf]

Supplementary Materials

Contents

Texts

Text S1. Adsorption features

Text S2. Preparation of tested biochars

Text S3. Determination of point of zero charge

Text S4. Determination of contact angle

Text S5. TCs measurements

Text S6. Adsorption micro-mechanisms

Text S7. Effects of environmental conditions

Tables

Table S1 Characteristic of the tested biochars

Table S2. The fitted parameters of PFO model and PSO model

Table S3. The fitted parameters for Langmuir model and Freundlich model

Table S4. The physicochemical properties and the quantum descriptors of tested TCs

Table S5. The valid adsorption energies of TCs of TCs with tested biochars

Table S6. The interaction types, distances and angels of TCs-biochars

Table S7. The standard curves and recovery rates of tested TCs

Figures

Figure S1. The influence of environmental factors (pH, Ca^{2+}, HA) on TTC and OTC adsorption efficiency (q_e) by tested biochars (a-f) and the statistical distribution of their adsorption efficiency (g).

Figure S2. C1s, O1s peaks of tested biochars by XPS regional spectra.

Figure S3. Equilibrium configurations of tested TCs (except TTC) adsorbed by PBC and GBC(a), adsorbed by py-PBC and py-GBC(b), adsorbed by pyr-PBC and pyr-GBC(c), adsorbed by gra-PBC and gra-GBC(d).

Figure S4 The scatterplots of reduced density gradients versus sign(λ2)ρ for the equilibrium configurations of TTC-biochars.

Figure S5. The redundancy analysis (RDA) of quantum chemical descriptors and E_{CVB}, E_{vdWs}, and $E_{es.}$

Total:

number of pages: 56

number of Texts: 7

number of Tables: 7

number of Figures: 5

Texts

Text S1. Adsorption features

The Langmuir model equation (Eq. S1) and the Freundlich model equation (Eq. S2) were employed to fit the adsorption isothermal data and evaluate the adsorption capacity of TCs on biochars, along with the associated interactions.

$$q_e = \frac{q_m \times K_L \times C_e^r}{1 + K_L \times C_e^r} \qquad \text{(Eq. S1)}$$

$$q_e = K_f \times (C_e^r)^{\frac{1}{n}} \qquad \text{(Eq. S2)}$$

where q_m (mg·g^{-1}) and K_L (L·mg^{-1}) represent the maximum capacity and the Langmuir constant, respectively, K_f is the Freundlich constant, and n is the parameter indicating the heterogeneity of the adsorbent surface.

In addition, an investigation into the adsorption kinetics of biochars in relation to TCs was undertaken. The experiment involved introducing 20 mg of biochars into a solution containing TCs with a concentration of 60 mg·L^{-1}. The adsorption process was analyzed at different time intervals, ranging from 10 to 1440 minutes. The obtained adsorption kinetics data were then fitted into the pseudo-first-order (PFO) model (Eq. S3) and pseudo-second-order (PSO) model (Eq. S4) using the following equations:

$$q_t = q_m - q_m \times \exp\left(-k_f \times t\right) \qquad \text{(Eq. S3)}$$

$$q_t = \frac{t}{\frac{1}{k_s \times q_m^2} + \frac{t}{q_m}} \qquad \text{(Eq. S4)}$$

where q_t is the adsorption capacity (mg·g^{-1}) at time t (min), q_m represents the maximum adsorption capacity. k_f and k_s are the kinetic rate constants (min^{-1}) of PFO and PSO models, respectively.

Moreover, the influence of solution characteristics on the adsorption process was investigated by utilizing 20 mg of biochar and 100 mL of TCs solution (with a concentration of 60 $mg \cdot L^{-1}$) in 250 mL brown glass vials. The pH values (ranging from 3 to 11), HA concentrations (ranging from 0 to 30 $mg \cdot L^{-1}$), and salinity concentrations (ranging from 0 to 10 $mg \cdot L^{-1}$) varied in the experiments. It is worth noting that the adsorption experiments were conducted in triplicate, and control groups were established to evaluate the impact of the adsorbent's absence.

Text S2. Preparation of tested biochars

To prepare the pristine biochar (PBC), the rice straw was cleaned, dried, and pulverized. In detail, the pulverized powder was then placed into a tube furnace and heated. The heating rate was 5 $°C \cdot min^{-1}$, the pyrolysis temperature was 700 °C, and the temperature was maintained for 2 h in a N_2 atmosphere. The N-doping pristine biochars (N-PBC) were synthesized through a direct pyrolysis process. In brief, rice straw-derived biochar (15 g) was mixed with $C_2H_4N_4$ (15 g) in deionized water (500 mL), followed by one hour of stirring, one hour of sonication, and a four-hour heating process at 80°C. Finally, the biomass was prepared in an N_2 environment, utilizing the same heating conditions as those used for biochar preparation.

To synthesize graphene-like biochar (GBC), a comprehensive process was implemented. (1) Rice straw-derived biochar (1 g) was mixed with K_2CO_3 (3 g) and ground for 20 minutes at an ambient temperature of approximately 25°C. The resulting mixture was then transferred to a corundum boat and heated in a tube furnace under the same conditions used for preparing biochar. (2) Before pyrolysis, high-purity N_2 (99.9%) was introduced into the system at a flow rate of 100 mL/min for one hour to purge the internal air. Subsequently, the temperature was ramped up to 600°C at a rate of 5°C/min under a N_2 environment and maintained for one hour. The

system was then cooled to room temperature. (3) The biochar obtained from step (2) was mixed with 50 mL of 1 M hydrochloric acid and shaken for 24 hours. The resulting mixture was washed with deionized water until a constant pH was achieved. The material was then dried at 60°C in a vacuum oven for 24 hours to yield GBC biochar. The process for generating N-doped graphene-like biochar (N-GBC) was carried out using GBC and following a similar preparation method as that of N-PBC. Under the specified preparation conditions, the biochar yields exhibit a range of 25-30%.

Text S3. Determination of point of zero charge

A 0.01 $mol \cdot L^{-1}$ sodium chloride solution was prepared in a beaker and adjusted to pH levels of 3, 4, 5, 6, 7, 8, and 9 as the initial pH values. Following this, 0.03 grams of the substance were precisely weighed and introduced into a 20 mL volumetric glass bottle. Subsequently, 10 mL aliquots of the aforementioned solutions at each pH were introduced into the bottle. The resultant mixture was agitated on a shaker for 48 hours at 25°C and 180 revolutions per minute (rpm). The pH was then measured upon achieving equilibrium, representing the equilibrium pH. The ΔpH (the difference between equilibrium and initial pH) values were then plotted against the initial pH values. The initial pH at which ΔpH is zero was taken to be the PZC [1].

Text S4. Determination of contact angle

The determination of the contact angle on the biochars surface employed the drop volume method. In detail, the biochars was positioned on a planar substrate, and an initial volume of water droplet was dispensed. Sequentially, incremental additions of water droplets were introduced at a controlled rate, facilitating continuous expansion. The dynamic evolution of the contact angle throughout this progression was quantified utilizing a contact angle measurement apparatus. The instrument used

was an optical contact angle meter (JCD2000D3M, Shanghai Zhongchen Digital Technic Apparatus CO., Ltd.) [2].

Text S5. TCs measurements

The concentration of TCs was assessed using an HPLC system (Shimadzu, LC-20AD, Japan) coupled with a C^{18} reverse-phase column. (250 mm × 4.6 mm, 5 μm, Shimadzu) maintained at a temperature of 28 °C. The mobile phases utilized consisted of oxalic acid (0.01 mol·L^{-1}), acetonitrile, and methanol in a ratio of 60%:20%:20% (v:v:v), and the flow rate was set at 1.0 mL·min^{-1}. The UV detector at a wavelength of 355 nm was employed to detect TCs, while the quantification principle for all samples was based on the utilization of the external standard HPLC method. Details regarding the standard curves and recovery rates of TCs can be found in Table S2.

Text S6. Adsorption micro-mechanisms

The adsorption energy (E_{ad}) of biochar-TCs was determined using the equation given below:

$$E_{ad} = E_{biochar-TC} - E_{biochar} - E_{TC} \qquad \text{(Eq. S5)}$$

where E_{ad} (KJ·mol^{-1}) represents the adsorption binding energy of biochar-TC; $E_{biochar-TC}$ (KJ·mol^{-1}), E_{TC}(KJ·mol^{-1}), and $E_{biochar}$ (KJ·mol^{-1}) represent the energies of biochar-TC, TC, and biochar, respectively.

The quantitative evaluation of the adsorption energy of TC on the prepared N-doped biochars was carried out by calculating the valid adsorption energy using Eq. S6. This calculation was based on the varying contents of the N functional groups present in the N-doped biochars. Table S6 lists the adsorption energies of TCs with tested biochars (E_{ad}) and the valid adsorption energy (E_{ad-v})

$$E_{ad-v} = \sum \varphi_{N^i} \times E_{ad^i} \quad \text{(Eq. S6)}$$

where φ_{N^i} represents the proportion of pyridinic-nitrogen, pyrrolic-nitrogen and graphitic-nitrogen of prepared N-doped biochars, and $E_{ad}{}^{i}$ (KJ·mol^{-1}) denotes the adsorption energy of N^i on biochar.

Based on the steady adsorption state of TCs on tested adsorbents, we also utilized BIOVIA Discovery Studio software 2017 (Accelrys) to interpret the microscopic mechanisms of the adsorption process [3]. We analyzed the adsorption equilibrium configurations and interaction features, such as interaction types, bond angle, and bond distance. Additionally, we employed the independent gradient model based on Hirshfeld partition (IGMH) in the main function 20 of the Multiwfn (version 3.8) program and Gaussian 09W software with B3LYP/6-31G(d) to illustrate the interactions between biochars and TCs [4]. Furthermore, the quantitative evaluation of the strength of various interactions (such as hydrogen bonding, π-π interactions, van der Waals forces(vdWs), and electrostatic interactions) between biochars and TCs was carried out using Multiwfn software, version 3.8 [5]. The aforementioned interactions were described in detail using several indices. Specifically, the core valence bifurcation (CVB) index was employed to quantify the characteristics of hydrogen bonding energy in the main function 200 of the Multiwfn (version 3.8) program. The features of vdWs forces were characterized by the vdWs interaction energies (E_{vdWs}) obtained from the EDA based on force field. The strength of electrostatic interactions (E_{es}) was quantitatively described using the electrostatic binding energies of EDA based on force filed [5].

Text S7. Effects of environmental conditions

To further verify the aforementioned conclusion, the impact of solution properties on the adsorption of TCs by the tested BCs was assessed under conditions of varying pH values (3-11), HA concentrations (0-30 mg·L^{-1}), and salinity

concentrations (0-10 mg·L^{-1}). Results presented in Fig.S1 a-b indicated that, the adsorption of TTC and OTC onto the tested BCs was significantly influenced by solution pH (P<0.01). Specifically, the q_e values of TTC and OTC ranged from 0.280 mg·g^{-1} to 1.86 mg·g^{-1}, depending on pH. The adsorption efficiency of TCs onto P-BC and GBC increased with increasing pH, while the adsorption efficiency of TCs onto N-BC and N-GBC increased from pH 3 to 9, but then declined from pH 9 to 11. Moreover, the removal capacity of two TCs onto P-BC was consistently greater than that onto N-BC across the tested pH range, while the removal capacity of TTC and OTC onto N-GBC was consistently greater than that onto GBC. In general, pH is a crucial environmental factor for adsorption and may considerably influence the surface charge of biochar and the ionization degree of pollutants. For example, the pKa values of TTC is 3.30 ($pKa1_{TC}$), 7.68 ($pKa2_{TC}$), and 9.69 ($pKa3_{TC}$). When solution pH was lower than pKa1, between pKa1 and pKa2, and higher than pKa2, TTC is mainly presented as protonated (H_3TC^+ and H_3OTC^+) and molecular states (H_2TC^0 and H_2OTC^0). Therefore, the observed effects of pH on TCs adsorption by the tested BCs may have been due to the electrostatic interaction between TCs species and the charged surface of BCs.

Changes in salinity can significantly impact the electrostatic interactions between chemicals and BCs, subsequently altering their adsorption features [6]. As shown in Fig. S1 c-d, our study found that the addition of Ca^{2+} had no significant effects on the adsorption of OTC-GBC (P>0.05), but did have significant effects on other adsorption processes (P<0.01 or P<0.05). The q_e values of OTC-GBC and OTC-BC ranged from 69.085 to 78.783 mg·g^{-1} and 47.958 mg·g^{-1} to 77.237 mg·g^{-1}, respectively. These results suggest that electrostatic interactions play a critical role in the adsorption of TCs onto most tested biochars. Additionally, our findings demonstrated that the

N-GBC exhibited the greatest removal rate of TCs under different concentrations of salinities, while the removal of TCs onto the PBC was consistently greater than that onto the N-BC. For example, the q_e values of TTC-PBC, TTC-N-BC, TTC-GBC, and TTC-N-GBC were 56.637 mg·g^{-1} to 72.570 mg·g^{-1}, 28.119 mg·g^{-1} to 36.912 mg·g^{-1}, 92.346 mg·g^{-1} to 103.444 mg·g^{-1}, and 151.190 mg·g^{-1} to 193.025 mg·g^{-1}, respectively. Therefore, the results also strongly proved that electrostatic interactions between TCs and biochars can be changed by the N doping process to varying degrees for PBC and GBC.

Humic acid (HA) is a typical dissolved organic matter with π electrons, aromatic rings, and fatty acids. As presented in Fig.S1 e~f, the HA has significant effects on the adsorption of tested TCs (OTC, TTC) ($P<0.01$). With the addition of HA (0~30 mg·L^{-1}), the removal rate of TCs for P-BC, N-BC, GBC, and N-GBC is increased 60.013%~111.611%, 229.966%~505.587%, 61.739%~107.160%, 22.232%~25.168%, respectively. It was speculated that the hydroxyl, keto and amino groups of TCs created bonds with the corresponding groups of HA, then the formed TCs-HA with extra adsorption sites played a "bridging" role to improve the adsorption capacity of TCs. π-π EDA interaction was an important mechanism of TCs adsorption by the tested BCs. Furthermore, among the tested biochars, N-GBC shows the highest removal efficiency at tested HA concentrations, and the removal of TCs onto PBC was consistently greater than that onto N-BC. For example, the mean removal efficiency of TTC by PBC, N-PBC, GBC, and N-GBC is 68.751 mg·L^{-1}, 35.313 mg·L^{-1}, 132.219 mg·L^{-1}, and 220.615 mg·L^{-1}, respectively. Thus, the results also proved that π-π EDA interactions between TCs and biochars were changed by the N doping process to varying degrees for PBC and GBC.

Overall, results of solution properties indicate that, (1) the significant

strengthening and complex effects of N doping on the TCs adsorption performance can be obtained for GBC and PBC, respectively; and (2) the electrostatic interaction and π-π interaction are important mechanisms of TCs.

Table S1 Characteristic of the tested biochars

Biochars	PBC	N-PBC	GBC	N-GBC
SSA_{BET} ($m^2 \cdot g^{-1}$)	374.670	203.110	807.410	935.090
V_t ($cm^3 \cdot g^{-1}$)	0.127	0.031	0.229	0.294
L_a (nm)	3.122	3.611	4.931	4.147
C%	50.590	48.470	42.900	45.160
H%	1.430	1.170	2.380	2.402
O%	2.050	11.442	10.640	19.978
N%	0.100	9.430	0.490	4.530
S%	0.000	0.0260	0.000	0.039
H/C	0.339	0.290	0.666	0.638
O/C	0.030	0.177	0.186	0.332
(O+N)/C	0.032	0.344	0.196	0.418
pH_{PZC}	9.86	8.62	5.96	6.15

Note: V_t presents the total pore volume of tested biochars; L_a presents the average pore diameter of tested biochars.

Table S2. The fitted parameters of PFO model and PSO model

TCs	Biochars	PFO			PSO		
		$K(10^{-2})$	q_e/(mg·g^{-1})	R^2	$K(10^{-3})$	q_e/(mg·g^{-1})	R^2
TTC	PBC	4.53	37.59	0.88	4.27	40.27	0.95
	N-PBC	0.52	16.96	0.97	0.05	20.91	0.98
	GBC	3.07	73.77	0.81	23.13	79.30	0.91
	N-GBC	1.89	152.06	0.88	123.72	165.70	0.95
OTC	PBC	4.03	38.73	0.87	4.16	41.16	0.95
	N-PBC	0.31	20.65	0.93	0.060	25.32	0.96
	GBC	2.45	78.13	0.80	21.70	83.61	0.90
	N-GBC	1.63	159.31	0.88	121.03	172.42	0.95
CTC	PBC	0.38	194.25	0.96	0.02	235.51	0.98
	N-PBC	0.52	224.68	0.95	0.02	263.20	0.97
	GBC	1.69	211.56	0.86	0.01	226.70	0.91
	N-GBC	0.45	237.33	0.92	0.02	276.94	0.95
DMC	PBC	5.75	54.94	0.88	1.48	57.94	0.92
	N-PBC	0.76	25.67	0.93	0.34	28.96	0.95
	GBC	10.16	141.81	0.94	1.21	146.81	0.96
	N-GBC	1.25	178.34	0.94	0.08	196.76	0.97
DOX	PBC	0.78	54.28	0.87	0.20	59.31	0.91
	N-PBC	4.51	17.05	0.81	4.19	17.80	0.79
	GBC	7.83	122.52	0.96	1.04	127.34	0.96
	N-GBC	2.36	148.07	0.95	0.24	156.78	0.97

MC	PBC	0.43	62.34	0.82	0.08	71.06	0.87
	N-PBC	0.39	25.80	0.93	0.12	31.95	0.96
	GBC	1.85	126.46	0.93	0.20	135.91	0.92
	N-GBC	3.74	140.78	0.88	0.39	148.99	0.93
MN	PBC	0.23	105.67	0.95	0.02	135.81	0.96
	N-PBC	0.20	190.67	0.96	0.02	257.68	0.99
	GBC	0.57	260.95	0.99	0.01	302.19	0.97
	N-GBC	0.61	283.72	0.95	0.02	327.20	0.97
TG	PBC	0.22	113.57	0.71	0.02	125.06	0.75
	N-PBC	0.15	195.59	0.96	0.01	270.25	0.97
	GBC	0.30	247.53	0.97	0.01	308.09	0.98
	N-GBC	0.45	249.89	0.99	0.02	299.16	0.99

Table S3 The fitted parameters for Langmuir model and Freundlich model

TCs	Biochars	Langmuir			Freundlich		
		Q_{max}/(mg·g^{-1})	K_L/(L·mg^{-1})	R^2	K_F /(mg^{1-n}·L^n·g^{-1})	n	R^2
TTC	PBC	60.26	0.87	0.9	31.76	6.25	0.86
	N-PBC	32.82	2.66	0.83	20.68	7.50	0.88
	GBC	82.58	1.25	0.74	38.66	4.98	0.89
	N-GBC	201.63	0.81	0.91	82.00	4.21	0.80
OTC	PBC	63.34	0.86	0.67	36.03	7.10	0.99
	N-PBC	32.80	0.33	0.71	13.14	4.60	0.95
	GBC	77.86	16.13	0.75	47.05	6.92	0.93
	N-GBC	193.04	11.25	0.92	113.83	6.16	0.96
CTC	PBC	193.69	0.14	0.99	46.09	2.97	0.94
	N-PBC	324.71	0.08	0.99	44.28	2.13	0.93
	GBC	291.95	0.59	0.97	105.99	3.42	0.86
	N-GBC	326.47	0.63	0.97	122.37	3.54	0.82
DMC	PBC	66.71	0.11	0.79	17.43	3.47	0.71
	N-PBC	51.55	0.04	0.86	5.14	2.18	0.78
	GBC	180.73	0.18	0.9	48.11	3.20	0.79
	N-GBC	220.84	0.17	0.89	57.29	3.14	0.75
DOX	PBC	76.53	0.17	0.87	23.91	3.78	0.97
	N-PBC	95.11	0.02	0.96	4.91	1.73	0.91
	GBC	160.59	0.38	0.88	57.12	3.900	0.79
	N-GBC	160.84	0.36	0.89	53.06	3.65	0.85
MC	PBC	106.84	0.11	0.98	24.22	3.07	0.88
	N-PBC	171.5	0.03	0.97	10.98	1.80	0.92
	GBC	170.18	0.16	0.91	40.58	2.97	0.85
	N-GBC	233.76	0.14	0.92	49.92	2.74	0.83

MN	PBC	115.25	0.09	0.99	21.02	2.66	0.96
	N-PBC	309.29	0.06	0.98	33.08	1.95	0.91
	GBC	321.71	0.16	0.91	69.83	2.6	0.77
	N-GBC	434.28	0.24	0.66	107.35	2.51	0.57
TG	PBC	552.26	0.02	0.91	22.5	1.55	0.86
	N-PBC	464.81	0.04	0.88	29.53	1.62	0.81
	GBC	491.11	0.08	0.92	64.61	1.98	0.84
	N-GBC	942.71	0.07	0.63	79.92	1.49	0.58

Table S4. The physicochemical properties and the quantum descriptors of tested TCs

	CTC	DMC	MC	MN	OTC	TTC	DOX	TG
Log K_{ow}[a]	-0.680	-1.140	-1.370	0.050	-0.900	-1.300	-0.020	-0.200
E_{homo}	-0.216	-0.383	-0.345	-0.336	-0.277	-0.340	-0.272	-0.336
E_{lumo}	-0.073	-0.057	-0.032	-0.045	-0.048	-0.058	-0.050	-0.054
ZPE	257.405	281.834	275.475	267.265	307.441	284.436	423.595	281.492
G	-1984.000	-1563.660	-2023.260	-1562.460	-1583.060	-1638.870	-2003.490	-1563.660
S	183.402	184.964	190.047	180.186	193.251	186.997	249.851	183.593
μ	3.775	5.079	4.057	6.081	6.725	6.464	8.484	5.885
E	-1984.350	-1564.050	-2023.640	-1562.830	-1583.490	-1639.270	-2004.090	-1564.050
q-	-0.281	-0.313	-0.313	-0.313	-0.314	-0.313	-0.312	-0.313
q^{H}+	0.184	0.167	0.160	0.156	0.157	0.171	0.158	0.161
V	289.521	290.115	301.488	284.051	308.368	293.460	409.279	289.711
$\varepsilon\alpha$	5.952	5.761	5.748	5.769	5.855	5.848	5.699	5.840

	CTC	DMC	MC	MN	OTC	TTC	DOX	TG
ATSC5p	-3.753	-5.539	-7.938	-5.105	-5.191	-6.311	-4.100	-5.233
MATS5p	-0.081	-0.113	-0.152	-0.112	-0.106	-0.126	-0.068	-0.108
GATS5p	1.031	1.083	1.137	1.079	1.101	1.099	1.084	1.086
GATS6i	1.007	1.017	1.026	1.037	1.027	1.011	1.019	1.023
SpMin1_Bhs	2.032	2.052	2.048	2.058	2.059	2.039	2.061	2.051
SHBint6	28.117	23.672	25.118	24.592	23.146	26.769	23.071	26.688
XLogP	-0.191	0.432	0.226	-0.009	0.342	-0.900	0.603	0.130
nCl	1.000	0.000	1.000	0.000	0.000	0.000	0.000	0.000
MATS4e	0.090	0.161	0.110	0.160	0.158	0.148	0.169	0.110
SpMax1_Bhv	3.987	3.990	3.999	3.996	3.985	3.994	3.990	3.992
SsCl	0.255	0.000	0.234	0.000	0.000	0.000	0.000	0.000
minHBa	0.255	1.372	0.234	1.342	1.522	1.293	1.498	1.442
minHBint7	0.061	0.620	0.049	0.753	0.400	0.618	0.290	1.286
MAXDP2	6.265	6.317	6.407	6.276	6.493	6.348	7.063	6.314

	CTC	DMC	MC	MN	OTC	TTC	DOX	TG
Zagreb	184.000	184.000	192.000	184.000	188.000	192.000	234.000	186.000
ATSC6p	-2.153	-3.437	-3.045	-3.775	-3.460	-3.472	-2.777	-3.266
MATS5p	-0.081	-0.113	-0.152	-0.112	-0.106	-0.126	-0.068	-0.108
SpMin1_Bhv	2.092	2.100	2.098	2.095	2.098	2.099	2.102	2.100
AATSC1m	-2.52	-1.690	-2.217	-1.982	-0.623	-2.255	-0.275	-1.690
ETA_Shape_X	0.034	0.034	0.066	0.035	0.033	0.068	0.052	0.069
MDEC-33	33.064	34.903	28.230	34.903	28.230	30.050	33.140	25.255
AMW	8.757	7.931	8.538	8.188	7.620	8.073	7.226	7.931
AATS6m	63.744	54.462	60.797	58.321	53.423	56.514	56.770	56.444

Note: [a] values of K_{ow} were obtained from EPIWEB 4.0; E_{homo} represents energy of the highest occupied molecular orbital (eV); E_{lumo} represents energy of the lowest unoccupied molecular orbital(eV); ZPE represents zero-point energy; *G* represents gibbs free energy; *S* represents entropy, μ represents dipole momen; *E* represents total electronic energy; *q*- represents most negative atomic charge; $q^{\mathrm{H+}}$ represents most positive atomic charge on the H atom; *V* represents molecular volume; $\varepsilon\alpha$ represents covalent acidity; *ATSC5p* representscentered broto-moreau autocorrelation-lag5/weighted by polarizabilities; *MATS5p* represents moran autocorrelation-lag5/weighted by polarizabilities; *GATS5p* represents geary autocorrelation-lag 5/weighted by polarizabilities; *GATS6i* represents Geary autocorrelation-lag6/weighted by first ionization potential; *SpMin1_Bhs* represents smallest absolute eigenvalue of burden modified matrix -n1/weighted by relative

I-state; *SHBint6* represents sum of E-State descriptors of strength for potential hydrogen bonds of path length 6; *XlogP* represents XlogPDescriptor; *nCl* represents number of chlorine atoms; *MATS4e* represents moran autocorrelation - lag 4 / weighted by sanderson electronegativities; *SpMax1_Bhv* represents largest absolute eigenvalue of burden modified matrix-n1/weighted by relative van der Waals volumes; *SsCl* resprsents sum of atom-type E-State:-Cl; *minHBint7* represnets minimum e-state descriptors of strength for potential hydrogen bonds of path length 7; *MAXDP2* represnts maximum positive intrinsic state difference in the molecule; *Zagreb* represents sum of the squares of atom degree over all heavy atoms *I*; *ATSC6p* represents centered broto-moreau autocorrelation - lag 6/weighted by polarizabilities; *SpMin1_Bhv* represents smallest absolute eigenvalue of burden modified matrix - n 1 / weighted by relative van der Waals volumes; *AATSC1m* represents average centered Broto-Moreau autocorrelation - lag 1 / weighted by mass; *ETA_Shape_X* means shape index X; *MDEC-33* represents molecular distance edge between all tertiary carbons; *AMW* denotes average molecular weight (Molecular weight / Total number of atoms); *AATS6m* denotes average broto-moreau autocorrelation - lag 6 / weighted by mass; *GATS5p* represents geary autocorrelation - lag 5 / weighted by polarizabilities.

Table S5. The valid adsorption energies ($KJ\cdot mol^{-1}$) of TCs with tested biochars ($E_{ad\text{-}v}$)

	DOX	DMC	CTC	MC	MN	TG	OTC	TTC
PBC	-1590.61	-1334.06	-1667.58	-1590.61	-1564.95	-1847.16	-1590.61	-1667.58
py-PBC	-1590.67	-1385.37	-1667.57	-1616.27	-1590.61	-1898.47	-1539.30	-1513.64
pr-PBC	-1616.26	-1385.39	-1693.23	-1616.26	-1590.60	-1898.46	-1564.96	-1641.92
gra-PBC	-1487.99	-1257.10	-1590.61	-1513.65	-1462.33	-1770.19	-2488.53	-1539.30
N-PBC [a]	-1578.88	-1359.14	-1661.09	-1595.28	-1564.38	-1872.24	-1742.67	-1565.16
GBC	-1641.92	-1411.03	-1744.54	-1667.58	-1616.27	-1975.43	-1539.30	-1590.61
py-GBC	-1616.27	-1385.36	-1718.88	-1616.28	-1590.62	-1924.13	-1513.64	-1564.96
pr-GBC	-1616.26	-1411.03	-1718.89	-1616.25	-1590.61	-1949.78	-1564.38	-1564.96
gra-GBC	-1641.92	-1411.02	-1744.54	-1641.92	-1616.26	-1949.79	-1513.65	-1590.60
N-GBC [a]	-1626.81	-1403.43	-1729.43	-1626.81	-1601.16	-1942.19	-1528.67	-1575.50

Note: [a] the valid adsorption energy ($E_{ad\text{-}v}$) that was calculated by Eq.S6.

Table S6. The interaction types, distances and angels of TCs-biochars

No.	Mixture	Type of interaction	Distance (Å)	Angle1(º)	Angle2(º)
1.	PBC-DOX	Pi-Alkyl	2.904	4.738	/
2.		Pi-Sigma	2.484	[c]10.392	[d]6.168
3.		Pi-Alkyl	4.527	/	/
4.		Pi-Alkyl	4.238	/	
5.		Pi-Pi Stacked	5.384	[d]40.360	[d]48.096
6.		Pi-Pi Stacked	5.218	[d]32.364	[d]46.435
7.		Pi-Pi Stacked	4.868	[d]45.098	[d]42.380
8.		Pi-Pi Stacked	3.850	[d]13.668	[d]20.937
9.		Pi-Pi Stacked	4.492	[d]25.750	[d]36.823
10.		Pi-Pi Stacked	4.080	[d]41.096	[d]28.200
11.		Pi-Pi Stacked	3.859	[d]28.849	[d]21.260
12.	PBC-DMC	Pi-Lone Pair	2.904	[d]10.331	
13.		Pi-Donor Hydrogen Bond	2.780	[c]19.111	[d]34.503
14.		Pi-Alkyl	5.088	/	/
15.		Pi-Alkyl	5.018	/	/
16.		Pi-Alkyl	5.293	/	/
17.		Pi-Pi Stacked	4.893	[d]33.841	[d]43.770
18.		Pi-Pi Stacked	4.767	[d]38.471	[d]42.155
19.		Pi-Alkyl	3.951	/	/
20.		Pi-Pi Stacked	4.621	[d]31.811	[d]40.124
21.		Pi-Alkyl	4.875	/	/
22.		Pi-Pi Stacked	3.608	[d]4.937	[d]11.667
23.		Pi-Pi Stacked	4.348	[d]41.392	[d]35.650
24.		Pi-Pi Stacked	4.040	[d]39.327	[d]28.994
25.		Pi-Pi Stacked	4.189	[d]34.366	[d]32.473
26.		Pi-Alkyl	4.300	/	/
27.	PBC-CTC	Pi-Alkyl	4.988	/	/
28.		Pi-Alkyl	4.687	/	/
29.		Pi-Alkyl	4.102	/	/
30.		Pi-Alkyl	4.587	/	/
31.	PBC-MC	Pi-Pi Stacked	4.476	[d]32.294	[d]47.119

No.	Mixture	Type of interaction	Distance (Å)	Angle1(º)	Angle2(º)
32.		Pi-Pi Stacked	4.612	[d]36.897	[d]48.674
33.		Pi-Alkyl	3.447	/	/
34.		Pi-Alkyl	4.109	/	/
35.		Pi-Alkyl	4.335	/	/
36.		Pi-Alkyl	5.366	/	/
37.	PBC-MN	Pi-Pi Stacked	4.426	[d]26.550	[d]40.247
38.		Pi-Pi Stacked	4.473	[d]35.516	[d]40.940
39.		Pi-Pi Stacked	5.935	[d]43.930	[d]55.300
40.		Pi-Alkyl	4.094	/	/
41.		Pi-Alkyl	5.478	/	/
42.		Pi-Alkyl	3.996	/	/
43.		Pi-Alkyl	4.701	/	/
44.		Pi-Alkyl	5.331	/	/
45.		Pi-Alkyl	4.724	/	/
46.		Pi-Alkyl	5.218	/	/
47.		Pi-Alkyl	4.664	/	/
48.	PBC-OTC	Pi-Pi Stacked	4.509	[d]48.117	[d]38.419
49.		Pi-Pi Stacked	3.685	[d]27.909	[d]16.512
50.		Pi-Pi Stacked	4.006	[d]17.309	[d]28.119
51.		Pi-Pi Stacked	3.960	[d]25.369	[d]26.871
52.		Pi-Pi Stacked	5.436	[d]52.189	[d]49.471
53.		Pi-Pi Stacked	5.027	[d]34.421	[d]45.350
54.		Pi-Pi Stacked	5.659	[d]46.421	[d]51.366
55.		Pi-Sigma	2.535	[c]7.394	/
56.	PBC-TG	Pi-Pi Stacked	4.078	[d]35.764	[d]26.700
57.		Pi-Pi Stacked	3.828	[d]20.044	[d]17.900
58.		Pi-Pi Stacked	5.017	[d]45.986	[d]43.432
59.		Pi-Pi Stacked	4.010	[d]19.784	[d]24.708
60.		Pi-Pi Stacked	4.607	[d]28.174	[d]37.7425
61.		Pi-Pi Stacked	5.633	[d]45.474	[d]49.703
62.		Pi-Pi Stacked	5.455	[d]39.029	[d]48.098
63.		Pi-Alkyl	4.925	/	/
64.		Pi-Alkyl	5.195	/	/

No.	Mixture	Type of interaction	Distance (Å)	Angle1(º)	Angle2(º)
65.		Pi-Alkyl	4.541	/	/
66.		Pi-Alkyl	4.030	/	/
67.		Pi-Alkyl	5.107	/	/
68.		Pi-Alkyl	4.440	/	/
69.		Pi-Alkyl	4.738	/	/
70.		Pi-Alkyl	5.352	/	/
71.		Pi-Alkyl	5.320	/	/
72.		Pi-Alkyl	4.564	/	/
73.	PBC-TTC	Pi-Pi Stacked	5.420	[d]33.728	[d]64.403
74.		Pi-Pi Stacked	5.855	[d]46.108	[d]63.905
75.		Pi-Pi Stacked	4.215	[d]27.766	[d]54.290
76.		Pi-Alkyl	4.783	/	/
77.		Pi-Alkyl	4.492	/	/
78.		Pi-Alkyl	3.879	/	/
79.		Pi-Alkyl	5.327	/	/
80.		Pi-Alkyl	4.278	/	/
81.		Pi-Donor Hydrogen Bond	3.175	[c]20.563	[d]27.549
82.	GBC-DOX	Pi-Pi Stacked	5.249	[d]30.091	[d]52.0544
83.		Pi-Pi Stacked	4.231	[d]7.219	[d]40.292
84.		Pi-Pi Stacked	4.742	[d]35.635	[d]47.118
85.		Pi-Alkyl	5.080	/	/
86.		Pi-Alkyl	5.323	/	/
87.		Pi-Alkyl	4.332	/	/
88.		Pi-Alkyl	5.331	/	/
89.		Pi-Alkyl	4.841	/	/
90.		Pi-Alkyl	5.095	/	/
91.		Pi-Pi Stacked	4.844	[d]24.217	[d]41.430
92.		Pi-Pi Stacked	3.708	[d]12.137	[d]11.539
93.		Pi-Pi Stacked	4.273	[d]48.489	[d]31.776
94.		Pi-Pi Stacked	4.425	[d]26.184	[d]34.826
95.		Pi-Pi Stacked	4.120	[d]39.526	[d]28.153
96.		Pi-Pi Stacked	5.348	[d]63.510	[d]47.215
97.		Pi-Pi Stacked	5.471	[d]49.891	[d]48.402

No.	Mixture	Type of interaction	Distance (Å)	Angle1(º)	Angle2(º)
98.		Pi-Pi Stacked	4.974	[d]35.0665	[d]43.090
99.		Pi-Pi Stacked	4.705	[d]45.834	[d]39.464
100.		Pi-Lone Pair	2.954	[d]8.369	/
101.	GBC-DMC	Pi-Alkyl	4.203	/	/
102.		Pi-Alkyl	4.983	/	/
103.		Pi-Alkyl	4.973	/	/
104.		Pi-Alkyl	4.010	/	/
105.		Pi-Pi Stacked	4.920	[d]35.846	[d]44.763
106.		Pi-Pi Stacked	4.311	[d]35.251	[d]35.870
107.		Pi-Alkyl	5.349	/	/
108.		Pi-Pi Stacked	3.853	[d]34.848	[d]24.949
109.		Pi-Pi Stacked	5.033	[d]56.151	[d]46.046
110.		Pi-Pi Stacked	4.091	[d]36.889	[d]31.356
111.		Pi-Alkyl	4.972	/	/
112.		Pi-Alkyl	4.977	/	/
113.		Pi-Pi Stacked	4.729	[d]37.734	[d]42.373
114.		Pi-Lone Pair	2.884	/	/
115.		Pi-Alkyl	5.359	/	/
116.		Pi-Pi Stacked	5.109	[d]36.662	[d]46.854
117.		Pi-Pi Stacked	3.655	[d]6.670	[d]17.085
118.		Pi-Alkyl	4.643	/	/
119.		Pi-Pi Stacked	5.215	[d]54.164	[d]47.951
120.		Pi-Alkyl	4.003	/	/
121.		Pi-Donor Hydrogen Bond	2.727	[d]30.242	[c]28.869
122.	GBC-CTC	Pi-Alkyl	5.080	/	/
123.		Pi-Alkyl	5.323	/	/
124.		Pi-Alkyl	4.332	/	/
125.		Pi-Alkyl	5.331	/	/
126.		Pi-Alkyl	4.841	/	/
127.		Pi-Alkyl	5.095	/	/
128.		Pi-Pi Stacked	4.974	[d]35.066	[d]43.090
129.		Pi-Pi Stacked	4.705	[d]45.834	[d]39.464
130.		Pi-Pi Stacked	4.844	[d]24.217	[d]41.430

No.	Mixture	Type of interaction	Distance (Å)	Angle1(º)	Angle2(º)
131.		Pi-Pi Stacked	3.708	d12.137	d11.539
132.		Pi-Pi Stacked	4.273	d48.489	d31.776
133.		Pi-Lone Pair	2.954	d8.369	/
134.		Pi-Pi Stacked	4.425	d26.184	d34.828
135.		Pi-Pi Stacked	4.120	d39.526	d28.153
136.		Pi-Pi Stacked	5.348	d63.510	d47.215
137.		Pi-Pi Stacked	5.471	d49.891	d48.402
138.	GBC-MC	Pi-Alkyl	5.177	/	/
139.		Pi-Alkyl	5.276	/	/
140.		Pi-Alkyl	4.471	/	/
141.		Pi-Alkyl	5.013	/	/
142.		Pi-Alkyl	5.008	/	/
143.		Pi-Alkyl	3.799	/	/
144.		Pi-Alkyl	4.245	/	/
145.		Pi-Alkyl	4.036	/	/
146.		Pi-Alkyl	3.572	/	/
147.		Pi-Alkyl	4.844	/	/
148.		Pi-Alkyl	4.662	/	/
149.		Pi-Alkyl	5.032	/	/
150.		Pi-Pi Stacked	5.363	d44.681	d50.236
151.		Pi-Pi Stacked	4.455	d31.710	d39.644
152.		Pi-Pi Stacked	5.016	d41.328	d46.857
153.		Pi-Pi Stacked	3.850	d25.233	d27.005
154.		Pi-Pi Stacked	3.609	d16.530	d18.084
155.		Pi-Pi Stacked	5.045	d47.064	d47.160
156.		Pi-Pi Stacked	3.887	d35.818	d28.055
157.		Pi-Pi Stacked	4.520	d46.180	d40.621
158.		Pi-Alkyl	5.437	/	/
159.		Pi-Alkyl	5.118	/	/
160.		Pi-Alkyl	5.098	/	/
161.		Pi-Alkyl	4.715	/	/
162.		Pi-Alkyl	5.398	/	/
163.		Pi-Alkyl	5.417	/	/

No.	Mixture	Type of interaction	Distance (Å)	Angle1(º)	Angle2(º)
164.		Pi-Alkyl	4.501	/	/
165.		Pi-Alkyl	5.040	/	/
166.		Pi-Pi Stacked	5.544	[d]43.509	[d]50.823
167.		Pi-Pi Stacked	5.303	[d]45.981	[d]48.672
168.		Pi-Alkyl	4.190	/	/
169.		Pi-Alkyl	3.947	/	/
170.		Pi-Pi Stacked	4.973	[d]36.595	[d]45.237
171.		Pi-Pi Stacked	3.874	[d]19.251	[d]25.299
172.		Pi-Pi Stacked	4.417	[d]41.238	[d]37.544
173.		Pi-Alkyl	5.392	/	/
174.		Pi-Pi Stacked	5.741	[d]46.321	[d]52.422
175.		Pi-Pi Stacked	4.014	[d]25.315	[d]29.265
176.		Pi-Pi Stacked	3.675	[d]26.343	[d]17.661
177.		Pi-Pi Stacked	5.013	[d]53.832	[d]45.687
178.		Pi-Pi Stacked	4.662	[d]45.534	[d]41.298
179.	GBC-MN	Pi-Alkyl	5.177	[d]55.109	[d]46.859
180.		Pi-Alkyl	5.177	/	/
181.		Pi-Alkyl	5.276	[a]156.703	[b]100.511
182.		Pi-Alkyl	4.471	/	/
183.		Pi-Alkyl	5.013	/	/
184.		Pi-Pi Stacked	4.662	[d]45.534	[d]41.298
185.		Pi-Alkyl	5.008	/	/
186.		Pi-Alkyl	3.799	/	/
187.		Pi-Alkyl	4.245	/	/
188.		Pi-Alkyl	4.036	/	/
189.		Pi-Alkyl	3.572	/	/
190.		Pi-Alkyl	4.844	/	/
191.		Pi-Alkyl	4.662	/	/
192.		Pi-Alkyl	5.032	/	/
193.		Pi-Pi Stacked	5.363	[d]44.681	[d]50.236
194.		Pi-Pi Stacked	4.455	[d]31.710	[d]39.644
195.		Pi-Pi Stacked	5.016	[d]41.328	[d]46.857
196.		Pi-Pi Stacked	3.850	[d]25.233	[d]27.005

No.	Mixture	Type of interaction	Distance (Å)	Angle1(º)	Angle2(º)
197.		Pi-Pi Stacked	3.609	[d]16.530	[d]18.084
198.		Pi-Pi Stacked	5.045	[d]47.064	[d]47.160
199.		Pi-Pi Stacked	3.887	[d]35.818	[d]28.055
200.		Pi-Pi Stacked	4.520	[d]46.180	[d]40.621
201.		Pi-Alkyl	5.437	/	/
202.		Pi-Alkyl	5.118	/	/
203.		Pi-Alkyl	5.098	/	/
204.		Pi-Alkyl	5.398	/	/
205.		Pi-Alkyl	5.417	/	/
206.		Pi-Alkyl	4.501	/	/
207.		Pi-Alkyl	5.040	/	/
208.		Pi-Pi Stacked	5.544	[d]43.509	[d]50.823
209.		Pi-Pi Stacked	5.303	d45.981	[d]48.672
210.		Pi-Alkyl	4.190	/	/
211.		Pi-Alkyl	3.947	/	/
212.		Pi-Pi Stacked	3.874	[d]19.251	[d]25.299
213.		Pi-Pi Stacked	4.417	[d]41.238	[d]37.544
214.		Pi-Alkyl	4.471	/	/
215.		Pi-Alkyl	5.013	/	/
216.		Pi-Pi Stacked	4.014	[d]25.315	[d]29.265
217.		Pi-Pi Stacked	3.675	[d]26.343	[d]17.661
218.		Pi-Pi Stacked	5.013	[d]53.832	[d]45.687
219.		Pi-Pi Stacked	4.662	[d]45.534	[d]41.298
220.		Pi-Pi Stacked	5.122	[d]55.109	[d]46.859
221.	GBC-OTC	Pi-Pi Stacked	5.935	[d]31.521	[d]57.685
222.		Pi-Pi Stacked	4.721	[d]36.765	[d]47.794
223.		Pi-Pi Stacked	4.119	[d]9.743	[d]39.635
224.		Pi-Pi Stacked	5.086	[d]31.501	[d]51.417
225.	GBC-TG	Pi-Alkyl	4.901	/	/
226.		Pi-Alkyl	4.367	/	/
227.		Pi-Alkyl	5.316	/	/
228.		Pi-Alkyl	5.325	/	/
229.		Pi-Alkyl	4.024	/	/

No.	Mixture	Type of interaction	Distance (Å)	Angle1(º)	Angle2(º)
230.		Pi-Alkyl	4.285	/	/
231.		Pi-Alkyl	5.248	/	/
232.		Pi-Alkyl	4.753	/	/
233.		Pi-Alkyl	5.437	/	/
234.		Pi-Alkyl	5.118	/	/
235.		Pi-Alkyl	4.715	/	/
236.		Pi-Alkyl	5.098	/	/
237.		Pi-Alkyl	5.398	/	/
238.		Pi-Alkyl	5.417	/	/
239.		Pi-Alkyl	4.501	/	/
240.		Pi-Alkyl	4.190	/	/
241.		Pi-Alkyl	5.392	/	/
242.		Pi-Alkyl	5.040	/	/
243.		Pi-Alkyl	3.947	/	/
244.		Pi-Alkyl	4.471	/	/
245.		Pi-Alkyl	5.276	/	/
246.		Pi-Alkyl	5.013	/	/
247.		Pi-Pi Stacked	5.544	[d]43.509	[d]50.823
248.		Pi-Pi Stacked	5.303	[d]45.981	[d]48.672
249.		Pi-Pi Stacked	4.973	[d]36.594	[d]45.237
250.		Pi-Pi Stacked	3.874	[d]19.251	[d]25.299
251.		Pi-Pi Stacked	4.417	[d]41.238	[d]37.544
252.		Pi-Pi Stacked	5.741	[d]46.321	[d]52.422
253.		Pi-Pi Stacked	4.014	[d]25.315	[d]29.265
254.		Pi-Pi Stacked	3.675	[d]26.343	[d]17.661
255.		Pi-Pi Stacked	5.013	[d]53.832	[d]45.687
256.		Pi-Pi Stacked	4.662	[d]45.534	[d]41.298
257.		Pi-Pi Stacked	5.122	[d]55.109	[d]46.859
258.		Pi-Alkyl	5.020	/	/
259.		Pi-Pi Stacked	4.644	[d]46.947	[d]39.748
260.		Pi-Pi Stacked	5.183	[d]48.427	[d]46.459
261.		Pi-Pi Stacked	5.153	[d]42.012	[d]46.143
262.		Pi-Pi Stacked	3.761	[d]18.819	[d]18.305

No.	Mixture	Type of interaction	Distance (Å)	Angle1(º)	Angle2(º)
263.		Pi-Pi Stacked	4.578	[d]30.360	[d]38.740
264.		Pi-Pi Stacked	3.995	[d]34.542	[d]26.655
265.		Pi-Pi Stacked	3.957	[d]21.524	[d]25.516
266.		Pi-Pi Stacked	5.439	[d]41.542	[d]48.964
267.		Pi-Pi Stacked	4.958	[d]46.524	[d]43.928
268.		Pi-Alkyl	5.375	/	/
269.		Pi-Pi Stacked	5.603	[d]47.274	[d]50.410
270.		Pi-Alkyl	4.735	/	/
271.		Pi-Alkyl	5.031	/	/
272.	GBC-TTC	Pi-Alkyl	4.652	/	/
273.		Pi-Alkyl	4.929	/	/
274.		Pi-Alkyl	4.274	/	/
275.		Pi-Alkyl	4.244	/	/
276.		Pi-Alkyl	4.643	/	/
277.		Pi-Alkyl	3.907	/	/
278.		Pi-Alkyl	4.927	/	/
279.		Pi-Alkyl	5.126	/	/
280.		Pi-Pi Stacked	5.902	[d]47.584	[d]58.290
281.		Pi-Pi Stacked	4.702	[d]38.560	[d]47.200
282.		Pi-Pi Stacked	4.613	[d]43.869	[d]43.914
283.		Pi-Pi Stacked	4.209	[d]32.454	[d]39.582
284.		Pi-Pi Stacked	3.400	[d]12.435	[d]16.320
285.		Pi-Pi Stacked	4.136	[d]43.228	[d]36.504
286.		Pi-Pi Stacked	3.648	[d]28.461	[d]25.631
287.		Pi-Pi Stacked	3.667	[d]35.050	[d]27.008
288.		Pi-Pi Stacked	5.010	[d]57.407	[d]48.512
289.		Pi-Pi Stacked	4.610	[d]51.141	[d]44.551
290.		Pi-Donor Hydrogen Bond	4.683	[d]39.742	[d]40.752
291.		Pi-Donor Hydrogen Bond	4.459	[d]37.282	[d]29.747
292.	pyr-PBC-DMC	Pi-Pi Stacked	4.482	[d]44.366	[d]37.665
293.		Pi-Pi Stacked	4.198	[d]41.174	[d]32.326
294.		Pi-Pi Stacked	3.580	[d]8.701	[d]7.775
295.		Pi-Pi Stacked	4.174	[d]32.633	[d]31.793

No.	Mixture	Type of interaction	Distance (Å)	Angle1(º)	Angle2(º)
296.		Pi-Pi Stacked	4.410	[d]28.037	[d]36.437
297.		Pi-Pi Stacked	5.073	/	/
298.		Pi-Pi Stacked	4.115	/	/
299.		Pi-Alkyl	4.321	/	/
300.		Pi-Alkyl	4.858	/	/
301.		Pi-Alkyl	5.207	/	/
302.		Pi-Alkyl	2.654	[a]113.791	[b]108.051
303.		Pi-Alkyl	2.059	[c]163.585	[d]130.513
304.		Conventional Hydrogen Bond	2.326	19.548	22.944
305.		Conventional Hydrogen Bond	3.928	[d]15.148	[d]23.470
306.		Pi-Donor Hydrogen Bond	3.799	[d]25.962	[d]18.499
307.	pyr-PBC-DOX	Pi-Pi Stacked	5.036	[d]44.311	[d]32.175
308.		Pi-Pi Stacked	4.448	[d]27.043	[d]35.897
309.		Pi-Pi Stacked	5.127	[d]45.109	[d]45.351
310.		Pi-Pi Stacked	4.459	/	/
311.		Pi-Pi Stacked	4.446	/	/
312.		Pi-Alkyl	4.292	/	/
313.		Pi-Alkyl	2.539	[c]2.887	[d]4.982
314.		Pi-Alkyl	5.021	/	/
315.		Pi-Sigma	4.174	/	/
316.	pyr-PBC-CTC	Pi-Alkyl	4.542	/	/
317.		Pi-Alkyl	4.359	/	/
318.		Pi-Alkyl	3.364	/	/
319.		Pi-Alkyl	4.021	/	/
320.	pyr-PBC-MC	Pi-Alkyl	4.224	/	/
321.		Pi-Alkyl	4.109	/	/
322.		Pi-Alkyl	5.335	/	/
323.		Pi-Alkyl	4.498	[d]32.889	[d]51.508
324.		Pi-Alkyl	4.817	[d]54.470	[d]40.591
325.		Pi-Pi Stacked	5.078	[d]34.921	[d]45.605
326.		Pi-Pi Stacked	3.943	[d]19.8845	[d]25.758
327.	pyr-PBC-MN	Pi-Pi Stacked	5.590	[d]42.636	[d]50.547
328.		Pi-Pi Stacked	5.391	[d]46.902	[d]48.794

No.	Mixture	Type of interaction	Distance (Å)	Angle1(º)	Angle2(º)
329.		Pi-Pi Stacked	4.475	/	/
330.		Pi-Pi Stacked	3.908	/	/
331.		Pi-Alkyl	4.451	/	/
332.		Pi-Alkyl	4.737	/	/
333.		Pi-Alkyl	5.351	/	/
334.		Pi-Alkyl	4.929	/	/
335.		Pi-Alkyl	4.635	/	/
336.		Pi-Alkyl	4.819	/	/
337.		Pi-Alkyl	2.162	[a]141.312	[b]94.076
338.		Pi-Alkyl	5.078	[d]34.921	[d]45.605
339.		Conventional Hydrogen Bond	3.943	[d]19.885	[d]25.758
340.	pyr-PBC-OTC	Pi-Pi Stacked	5.590	[d]42.636	[d]50.547
341.		Pi-Pi Stacked	5.391	[d]46.902	[d]48.794
342.		Pi-Pi Stacked	3.908	/	/
343.		Pi-Pi Stacked	4.475	/	/
344.		Pi-Alkyl	4.451	/	/
345.		Pi-Alkyl	4.737	/	/
346.		Pi-Alkyl	5.351	/	/
347.		Pi-Alkyl	4.635	/	/
348.		Pi-Alkyl	4.929	/	/
349.		Pi-Alkyl	4.819	/	/
350.		Pi-Alkyl	2.162	[a]141.312	[b]94.076
351.		Pi-Alkyl	4.093	[d]34.033	[d]26.679
352.		Conventional Hydrogen Bond	3.757	[d]9.417	[d]13.228
353.	pyr-PBC-TG	Pi-Pi Stacked	5.330	[d]48.618	[d]46.683
354.		Pi-Pi Stacked	4.314	[d]27.924	[d]32.023
355.		Pi-Pi Stacked	5.818	[d]51.067	[d]47.848
356.		Pi-Pi Stacked	4.853	[d]31.627	[d]41.105
357.		Pi-Pi Stacked	5.896	[d]44.359	[d]51.672
358.		Pi-Pi Stacked	4.509	/	/
359.		Pi-Pi Stacked	5.050	/	/
360.		Pi-Alkyl	4.323	/	/
361.		Pi-Alkyl	4.090	/	/

No.	Mixture	Type of interaction	Distance (Å)	Angle1(º)	Angle2(º)
362.		Pi-Alkyl	5.334	/	/
363.		Pi-Alkyl	4.699	/	/
364.		Pi-Alkyl	4.986	/	/
365.		Pi-Alkyl	5.057	/	/
366.		Pi-Alkyl	5.340	/	/
367.		Pi-Alkyl	4.920	/	/
368.		Pi-Alkyl	4.615	/	/
369.		Pi-Alkyl	2.034	[a]156.703	[b]100.511
370.		Pi-Alkyl	5.009	/	/
371.		Conventional Hydrogen Bond	4.379	/	/
372.	pyr-PBC-TTC	Pi-Alkyl	4.135	/	/
373.		Pi-Alkyl	4.085	/	/
374.		Pi-Alkyl	4.711	/	/
375.		Pi-Alkyl	2.910	[c]15.004	/
376.		Pi-Alkyl	2.614	[c]18.314	/
377.		Pi-Sigma	3.018	[d]32.233	/
378.		Pi-Sigma	5.043	[d]47.976	[d]45.974
379.		Pi-Donor Hydrogen Bond	5.008	[d]39.809	[d]45.565
380.	pyr-GBC-DMC	Pi-Pi Stacked	5.014	[d]49.192	[d]45.628
381.		Pi-Pi Stacked	5.920	[d]53.688	[d]44.127
382.		Pi-Pi Stacked	5.639	[d]48.1781	[d]51.535
383.		Pi-Pi Stacked	4.519	[d]48.048	[d]39.154
384.		Pi-Pi Stacked	3.657	[d]17.397	[d]16.567
385.		Pi-Pi Stacked	4.473	[d]27.820	[d]38.385
386.		Pi-Pi Stacked	5.451	[d]60.674	[d]49.995
387.		Pi-Pi Stacked	3.960	[d]37.457	[d]27.727
388.		Pi-Pi Stacked	3.945	[d]23.524	[d]27.317
389.		Pi-Pi Stacked	5.418	[d]40.900	[d]49.667
390.		Pi-Pi Stacked	4.643	/	/
391.		Pi-Pi Stacked	4.717	/	/
392.		Pi-Alkyl	5.378	/	/
393.		Pi-Alkyl	4.677	/	/
394.		Pi-Alkyl	4.049	/	/

No.	Mixture	Type of interaction	Distance (Å)	Angle1(º)	Angle2(º)
395.		Pi-Alkyl	4.075	/	/
396.		Pi-Alkyl	4.967	/	/
397.		Pi-Alkyl	4.211	/	/
398.		Pi-Alkyl	4.784	/	/
399.		Pi-Alkyl	2.953	[d]15.856	/
400.		Pi-Alkyl	1.937	[a]143.033	[b]127.798
401.		Pi-Lone Pair	5.138	[d]31.585	[d]43.934
402.		Conventional Hydrogen Bond	4.666	[d]22.687	[d]37.523
403.	pyr-GBC-DOX	Pi-Pi Stacked	3.822	[d]12.975	[d]14.559
404.		Pi-Pi Stacked	4.399	[d]32.729	[d]43.675
405.		Pi-Pi Stacked	5.645	[d]40.610	[d]49.040
406.		Pi-Pi Stacked	4.182	[d]28.366	[d]27.778
407.		Pi-Pi Stacked	4.111	[d]41.241	[d]25.837
408.		Pi-Pi Stacked	5.191	[d]54.169	[d]44.532
409.		Pi-Pi Stacked	4.356	/	/
410.		Pi-Pi Stacked	5.065	/	/
411.		Pi-Alkyl	4.007	/	/
412.		Pi-Alkyl	5.187	/	/
413.		Pi-Alkyl	5.031	/	/
414.		Pi-Alkyl	4.121	/	/
415.		Pi-Alkyl	4.746	/	/
416.		Pi-Alkyl	2.978	[d]7.831	/
417.		Pi-Alkyl	2.851	[d]6.172	/
418.		Pi-Lone Pair	2.726	[c]3.537	[d]17.826
419.		Pi-Lone Pair	4.881	[d]24.977	[d]41.709
420.		Pi-Sigma	4.356	[d]24.127	[d]33.242
421.	pyr-GBC-CTC	Pi-Pi Stacked	5.080	[d]44.200	[d]45.802
422.		Pi-Pi Stacked	5.073	[d]36.580	[d]44.083
423.		Pi-Pi Stacked	3.753	[d]13.453	[d]13.797
424.		Pi-Pi Stacked	4.050	[d]37.564	[d]25.888
425.		Pi-Pi Stacked	4.803	[d]46.661	[d]40.655
426.		Pi-Pi Stacked	4.266	[d]47.396	[d]31.332
427.		Pi-Pi Stacked	5.272	[d]62.525	[d]46.283

No.	Mixture	Type of interaction	Distance (Å)	Angle1(º)	Angle2(º)
428.		Pi-Pi Stacked	5.392	/	/
429.		Pi-Pi Stacked	5.359	/	/
430.		Pi-Alkyl	5.118	/	/
431.		Pi-Alkyl	4.338	/	/
432.		Pi-Alkyl	5.021	/	/
433.		Pi-Alkyl	4.784	/	/
434.		Pi-Alkyl	2.782	^{c}17.284	^{d}12.485
435.		Pi-Alkyl	5.360	^{d}48.269	^{d}50.486
436.		Pi-Sigma	5.047	^{d}47.487	^{d}49.353
437.	pyr-GBC-MC	Pi-Pi Stacked	4.542	^{d}46.907	^{d}41.333
438.		Pi-Pi Stacked	3.772	^{d}25.198	^{d}25.305
439.		Pi-Pi Stacked	4.700	^{d}37.533	^{d}43.483
440.		Pi-Pi Stacked	4.892	^{d}45.840	^{d}50.603
441.		Pi-Pi Stacked	4.484	^{d}40.975	^{d}40.510
442.		Pi-Pi Stacked	5.305	^{d}45.682	^{d}50.014
443.		Pi-Pi Stacked	5.381	^{d}43.894	^{d}50.678
444.		Pi-Pi Stacked	3.773	^{d}19.358	^{d}25.339
445.		Pi-Pi Stacked	3.663	^{d}27.743	^{d}21.388
446.		Pi-Pi Stacked	4.117	/	/
447.		Pi-Pi Stacked	3.653	/	/
448.		Pi-Alkyl	4.880	/	/
449.		Pi-Alkyl	4.764	/	/
450.		Pi-Alkyl	3.463	/	/
451.		Pi-Alkyl	3.910	/	/
452.		Pi-Alkyl	5.001	/	/
453.		Pi-Alkyl	4.606	/	/
454.		Pi-Alkyl	5.934	^{d}47.113	^{d}53.576
455.		Pi-Alkyl	5.480	^{d}47.935	^{d}50.013
456.	pyr-GBC-MN	Pi-Pi Stacked	5.353	^{d}40.354	^{d}48.854
457.		Pi-Pi Stacked	4.096	^{d}24.779	^{d}30.700
458.		Pi-Pi Stacked	4.330	^{d}39.180	^{d}35.574
459.		Pi-Pi Stacked	4.205	^{d}26.430	^{d}33.106
460.		Pi-Pi Stacked	3.572	^{d}17.572	^{d}9.6343

No.	Mixture	Type of interaction	Distance (Å)	Angle1(º)	Angle2(º)
461.		Pi-Pi Stacked	4.652	[d]49.086	[d]40.798
462.		Pi-Pi Stacked	5.749	[d]48.298	[d]52.196
463.		Pi-Pi Stacked	4.603	[d]41.319	[d]40.072
464.		Pi-Pi Stacked	4.804	[d]49.917	[d]42.845
465.		Pi-Pi Stacked	4.480	/	/
466.		Pi-Pi Stacked	4.407	/	/
467.		Pi-Alkyl	4.603	/	/
468.		Pi-Alkyl	3.866	/	/
469.		Pi-Alkyl	5.085	/	/
470.		Pi-Alkyl	4.889	/	/
471.		Pi-Alkyl	4.731	/	/
472.		Pi-Alkyl	5.009	/	/
473.		Pi-Alkyl	4.630	/	/
474.		Pi-Alkyl	4.956	/	/
475.		Pi-Alkyl	5.499	/	/
476.		Pi-Alkyl	4.902	/	/
477.		Pi-Alkyl	2.727	[c]18.647	[d]13.576
478.		Pi-Alkyl	2.228	[a]130.714	[b]94.936
479.		Pi-Sigma	5.169	[d]45.216	[d]51.946
480.		Conventional Hydrogen Bond	4.356	[d]29.364	[d]33.258
481.	pyr-GBC-OTC	Pi-Pi Stacked	4.773	[d]20.878	[d]40.246
482.		Pi-Pi Stacked	4.139	[d]44.428	[d]28.324
483.		Pi-Pi Stacked	3.706	[d]13.686	[d]10.565
484.		Pi-Pi Stacked	4.924	[d]30.349	[d]42.266
485.		Pi-Pi Stacked	5.445	[d]67.375	[d]47.986
486.		Pi-Pi Stacked	4.360	[d]51.029	[d]33.326
487.		Pi-Pi Stacked	4.749	[d]44.420	[d]39.896
488.		Pi-Pi Stacked	5.393	/	/
489.		Pi-Pi Stacked	5.427	/	/
490.		Pi-Alkyl	5.049	/	/
491.		Pi-Alkyl	4.365	/	/
492.		Pi-Alkyl	5.151	/	/
493.		Pi-Alkyl	4.837	/	/

No.	Mixture	Type of interaction	Distance (Å)	Angle1(º)	Angle2(º)
494.		Pi-Alkyl	2.898	^{d}5.760	/
495.		Pi-Alkyl	2.927	^{c}17.325	^{d}10.909
496.		Pi-Lone Pair	5.437	^{d}47.593	^{d}47.849
497.		Pi-Sigma	4.869	^{d}47.533	^{d}41.448
498.	pyr-GBC-TG	Pi-Pi Stacked	4.071	^{d}35.992	^{d}26.266
499.		Pi-Pi Stacked	3.921	^{d}20.332	^{d}21.384
500.		Pi-Pi Stacked	5.294	^{d}40.058	^{d}46.426
501.		Pi-Pi Stacked	4.843	^{d}46.795	^{d}41.065
502.		Pi-Pi Stacked	3.913	^{d}19.136	^{d}21.070
503.		Pi-Pi Stacked	4.620	^{d}28.042	^{d}37.804
504.		Pi-Pi Stacked	5.408	^{d}46.533	^{d}47.520
505.		Pi-Pi Stacked	5.322	^{d}39.573	^{d}46.680
506.		Pi-Pi Stacked	4.947	/	/
507.		Pi-Pi Stacked	4.792	/	/
508.		Pi-Alkyl	4.862	/	/
509.		Pi-Alkyl	5.369	/	/
510.		Pi-Alkyl	5.402	/	/
511.		Pi-Alkyl	4.101	/	/
512.		Pi-Alkyl	4.294	/	/
513.		Pi-Alkyl	4.971	/	/
514.		Pi-Alkyl	4.887	/	/
515.		Pi-Alkyl	4.323	/	/
516.		Pi-Alkyl	5.224	/	/
517.		Pi-Alkyl	2.694	^{c}38.183	^{d}13.251
518.		Pi-Alkyl	2.808	^{c}14.598	^{d}2.551
519.		Pi-Donor Hydrogen Bond	2.622	^{c}14.337	^{d}5.921
520.		Pi-Sigma	5.920	^{d}45.618	^{d}59.726
521.		Pi-Sigma	4.623	^{d}36.068	^{d}47.999
522.	pyr-GBC-TTC	Pi-Pi Stacked	4.279	^{d}31.856	^{d}41.162
523.		Pi-Pi Stacked	4.494	^{d}41.204	^{d}44.925
524.		Pi-Pi Stacked	3.355	^{d}8.600	^{d}12.829
525.		Pi-Pi Stacked	4.051	^{d}41.104	^{d}36.522
526.		Pi-Pi Stacked	3.828	^{d}30.468	^{d}31.198

No.	Mixture	Type of interaction	Distance (Å)	Angle1(º)	Angle2(º)
527.		Pi-Pi Stacked	3.776	d36.018	d30.645
528.		Pi-Pi Stacked	4.647	d45.655	d48.444
529.		Pi-Pi Stacked	4.337	/	/
530.		Pi-Pi Stacked	3.846	/	/
531.		Pi-Alkyl	5.295	/	/
532.		Pi-Alkyl	4.882	/	/
533.		Pi-Alkyl	4.538	/	/
534.		Pi-Alkyl	4.251	/	/
535.		Pi-Alkyl	4.913	/	/
536.		Pi-Alkyl	4.672	/	/
537.		Pi-Alkyl	3.029	c30.116	/
538.		Pi-Alkyl	2.879	c23.885	/
539.		Pi-Donor Hydrogen Bond	2.492	a137.608	b111.142
540.		Pi-Donor Hydrogen Bond	4.865	/	/
541.	py-PBC-DMC	Carbon Hydrogen Bond	1.762	a165.565	b104.728
542.		Pi-Alkyl	4.203	d35.155	d36.132
543.		Conventional Hydrogen Bond	4.675	d42.565	d43.430
544.		Pi-Pi Stacked	3.482	d12.767	d12.943
545.		Pi-Pi Stacked	4.837	d45.307	d45.520
546.		Pi-Pi Stacked	5.027	/	/
547.		Pi-Pi Stacked	3.838	d26.874	d24.190
548.		Pi-Alkyl	3.907	d25.990	d26.360
549.	py-PBC-DOX	Pi-Pi Stacked	4.543	d40.486	d39.746
550.		Pi-Pi Stacked	3.743	d18.998	d20.725
551.		Pi-Pi Stacked	4.653	d38.792	d41.278
552.		Pi-Pi Stacked	5.239	d45.848	d48.138
553.		Pi-Pi Stacked	5.196	d46.670	d47.679
554.		Pi-Pi Stacked	4.537	/	/
555.		Pi-Pi Stacked	5.167	/	/
556.		Pi-Alkyl	4.514	/	/
557.		Pi-Alkyl	2.593	c9.596	d11.706
558.		Pi-Alkyl	1.803	a155.337	b100.396
559.		Pi-Sigma	3.629	/	/

No.	Mixture	Type of interaction	Distance (Å)	Angle1(º)	Angle2(º)
560.		Conventional Hydrogen Bond	3.717	/	/
561.	py-PBC-CTC	Pi-Alkyl	4.882	/	/
562.		Pi-Alkyl	4.169	/	/
563.		Pi-Alkyl	5.021	/	/
564.		Pi-Alkyl	2.933	[d]9.545	/
565.		Pi-Alkyl	5.187	[d]44.361	[d]46.988
566.		Pi-Lone Pair	4.438	[d]39.754	[d]36.868
567.		Pi-Pi Stacked	5.366	[d]42.669	[d]48.690
568.		Pi-Pi Stacked	3.809	[d]16.412	[d]16.412
569.		Pi-Pi Stacked	3.803	[d]27.838	[d]21.100
570.		Pi-Pi Stacked	4.848	[d]36.608	[d]43.020
571.		Pi-Pi Stacked	4.048	[d]27.077	[d]28.854
572.		Pi-Pi Stacked	4.786	/	/
573.		Pi-Pi Stacked	4.161	/	/
574.		Pi-Alkyl	1.810	[a]164.523	[b]101.077
575.		Pi-Alkyl	4.117	/	/
576.		Conventional Hydrogen Bond	5.455	/	/
577.	py-PBC-MC	Pi-Alkyl	3.403	/	/
578.		Pi-Alkyl	4.155	/	/
579.		Pi-Alkyl	4.359	/	/
580.		Pi-Alkyl	4.501	[d]33.425	[d]48.637
581.		Pi-Alkyl	4.617	[d]36.688	[d]49.823
582.		Pi-Pi Stacked	4.640	[d]28.709	[d]43.881
583.		Pi-Pi Stacked	4.490	[d]35.153	[d]41.768
584.	py-PBC-MN	Pi-Pi Stacked	4.210	/	/
585.		Pi-Pi Stacked	3.949	/	/
586.		Pi-Alkyl	4.839	/	/
587.		Pi-Alkyl	5.327	/	/
588.		Pi-Alkyl	4.719	/	/
589.		Pi-Alkyl	4.668	/	/
590.		Pi-Alkyl	3.660	[d]22.028	[d]14.379
591.		Pi-Alkyl	4.207	[d]42.342	[d]32.550

No.	Mixture	Type of interaction	Distance (Å)	Angle1(º)	Angle2(º)
592.	py-PBC-OTC	Pi-Pi Stacked	4.298	[d]23.494	[d]34.450
593.		Pi-Pi Stacked	3.958	[d]22.404	[d]26.353
594.		Pi-Pi Stacked	5.210	[d]49.520	[d]47.249
595.		Pi-Pi Stacked	5.272	[d]37.140	[d]47.785
596.		Pi-Pi Stacked	5.672	[d]46.192	[d]51.363
597.		Pi-Pi Stacked	2.531	[c]12.366	[d]5.798
598.		Pi-Pi Stacked	2.894	[a]109.760	[b]101.174
599.		Pi-Sigma	4.580	/	/
600.		Carbon Hydrogen Bond	4.615	/	/
601.	py-PBC-TG	Pi-Alkyl	4.039	/	/
602.		Pi-Alkyl	4.312	/	/
603.		Pi-Alkyl	4.547	/	/
604.		Pi-Alkyl	5.483	/	/
605.		Pi-Alkyl	5.314	[d]43.431	[d]47.006
606.		Pi-Alkyl	5.357	[d]50.232	[d]47.581
607.		Pi-Pi Stacked	4.654	[d]30.182	[d]38.922
608.		Pi-Pi Stacked	3.872	[d]22.064	[d]20.821
609.		Pi-Pi Stacked	4.749	[d]48.333	[d]40.496
610.		Pi-Pi Stacked	3.943	[d]17.897	[d]23.449
611.		Pi-Pi Stacked	3.992	[d]33.873	[d]24.993
612.		Pi-Pi Stacked	4.460	[d]29.342	[d]61.295
613.		Pi-Pi Stacked	4.930	[d]42.174	[d]60.488
614.	py-PBC-TTC	Pi-Pi Stacked	2.707	[c]8.401	/
615.		Pi-Pi Stacked	2.380	[c]9.910	/
616.		Pi-Sigma	2.458	/	/
617.		Pi-Sigma	5.323	/	/
618.		Conventional Hydrogen Bond	4.402	/	/
619.		Pi-Alkyl	4.260	/	/
620.		Pi-Alkyl	5.260	/	/
621.		Pi-Alkyl	3.952	/	/
622.		Pi-Alkyl	4.363	/	/
623.		Pi-Alkyl	4.817	/	/
624.		Pi-Alkyl	5.025	/	/

No.	Mixture	Type of interaction	Distance (Å)	Angle1(º)	Angle2(º)
625.	py-GBC-TC	Pi-Alkyl	4.880	/	/
626.		Pi-Alkyl	4.504	/	/
627.		Pi-Alkyl	3.829	/	/
628.		Pi-Alkyl	4.261	/	/
629.		Pi-Alkyl	4.438	/	/
630.		Pi-Alkyl	5.022	/	/
631.		Pi-Alkyl	4.928	/	/
632.		Pi-Alkyl	2.574	[a]131.019	[b]115.097
633.		Pi-Alkyl	2.951	[d]13.165	/
634.		Carbon Hydrogen Bond	2.799	[c]34.769	[d] 31.286
635.		Pi-Lone Pair	5.315	[d] 58.040	[d] 48.789
636.		Pi-Donor Hydrogen Bond	5.340	[d] 52.927	[d] 48.956
637.		Pi-Pi Stacked	3.801	[d] 30.394	[d] 22.844
638.		Pi-Pi Stacked	4.670605	[d] 37.919	[d] 41.409
639.		Pi-Pi Stacked	5.408	[d] 40.120	[d] 40.120
640.		Pi-Pi Stacked	5.424	[d] 39.367	[d] 49.460
641.		Pi-Pi Stacked	4.705	[d] 36.132	[d] 41.880
642.		Pi-Pi Stacked	5.380	[d] 50.951	[d] 49.298
643.		Pi-Pi Stacked	4.628	[d] 54.705	[d] 38.336
644.		Pi-Pi Stacked	3.871	[d] 25.899	[d] 20.410
645.		Pi-Pi Stacked	4.025	[d] 13.971	[d] 25.636
646.		Pi-Pi Stacked	2.942	[d] 9.328	/
647.		Pi-Pi Stacked	4.308	/	/
648.		Pi-Lone Pair	4.576	/	/
649.		Pi-Alkyl	4.562	/	/
650.	py-GBC-MC	Pi-Alkyl	4.270	/	/
651.		Pi-Alkyl	5.467	/	/
652.		Pi-Alkyl	5.266	/	/
653.		Pi-Alkyl	4.006	/	/
654.		Pi-Alkyl	3.361	/	/
655.		Pi-Alkyl	3.980	/	/
656.		Pi-Alkyl	4.497	[d] 37.913	[d] 40.762
657.		Pi-Alkyl	5.127	[d] 50.469	[d] 48.682

No.	Mixture	Type of interaction	Distance (Å)	Angle1(º)	Angle2(º)
658.		Pi-Pi Stacked	3.642	[d] 25.444	[d] 20.636
659.		Pi-Pi Stacked	5.134	[d] 54.311	[d] 48.455
660.		Pi-Pi Stacked	4.526	[d] 47.984	[d] 41.090
661.		Pi-Pi Stacked	3.815	[d] 29.264	[d] 26.708
662.		Pi-Pi Stacked	4.771	[d] 40.566	[d] 44.330
663.		Pi-Pi Stacked	5.451	[d] 44.570	[d] 51.280
664.		Pi-Pi Stacked	3.792	[d] 19.001	[d] 25.986
665.		Pi-Pi Stacked	5.334	[d] 43.869	[d] 50.347
666.		Pi-Pi Stacked	4.941	/	/
667.		Pi-Pi Stacked	5.238	/	/
668.		Pi-Alkyl	5.153	/	/
669.	py-GBC-MN	Pi-Alkyl	4.744	/	/
670.		Pi-Alkyl	4.848	/	/
671.		Pi-Alkyl	5.306	/	/
672.		Pi-Alkyl	3.973	/	/
673.		Pi-Alkyl	5.254	/	/
674.		Pi-Alkyl	4.725	/	/
675.		Pi-Alkyl	4.198	/	/
676.		Pi-Alkyl	5.212	/	/
677.		Pi-Alkyl	4.270	/	/
678.		Pi-Alkyl	5.116	[d] 55.208	[d] 46.771
679.		Pi-Alkyl	3.811	[d] 30.881	[d] 23.058
680.		Pi-Pi Stacked	5.441	[d] 57.312	[d] 49.936
681.		Pi-Pi Stacked	5.005	[d] 48.914	[d] 45.560
682.		Pi-Pi Stacked	4.137	[d] 4.137	[d] 32.037
683.		Pi-Pi Stacked	5.825	[d] 46.973	[d] 52.934
684.		Pi-Pi Stacked	4.841	[d] 35.064	[d] 43.536
685.		Pi-Pi Stacked	5.216	[d] 40.682	[d] 47.788
686.		Pi-Pi Stacked	4.961	[d] 43.445	[d] 45.095
687.		Pi-Pi Stacked	4.271	[d] 40.327	[d] 34.761
688.		Pi-Pi Stacked	3.706	[d] 13.024	[d] 18.843
689.		Pi-Pi Stacked	2.923	[d] 10.332	/
690.		Pi-Pi Stacked	4.817	[d] 46.010	[d] 40.764

No.	Mixture	Type of interaction	Distance (Å)	Angle1(º)	Angle2(º)
691.		Pi-Lone Pair	5.571	[d] 40.848	[d] 49.133
692.	py-GBC-OTC	Pi-Pi Stacked	4.012	[d] 14.900	[d] 25.119
693.		Pi-Pi Stacked	5.572	[d] 31.572	[d] 49.315
694.		Pi-Pi Stacked	4.806	[d] 23.848	[d] 40.858
695.		Pi-Pi Stacked	5.420	[d] 41.751	[d] 47.968
696.		Pi-Pi Stacked	3.908	[d] 25.563	[d] 21.651
697.		Pi-Pi Stacked	5.353	[d] 53.670	[d] 47.333
698.		Pi-Pi Stacked	4.652	[d] 55.258	[d] 38.632
699.		Pi-Pi Stacked	5.368	[d] 66.531	[d] 47.462
700.		Pi-Pi Stacked	3.920	[d] 39.922	[d] 22.134
701.		Pi-Pi Stacked	5.444	62.862	[d] 48.191
702.		Pi-Pi Stacked	5.229	/	/
703.		Pi-Pi Stacked	4.992	/	/
704.		Pi-Alkyl	4.708	/	/
705.	py-GBC-TG	Pi-Alkyl	4.622	/	/
706.		Pi-Alkyl	4.376	/	/
707.		Pi-Alkyl	4.028	/	/
708.		Pi-Alkyl	5.250	/	/
709.		Pi-Alkyl	5.031	/	/
710.		Pi-Alkyl	5.433	/	/
711.		Pi-Alkyl	4.145	/	/
712.		Pi-Alkyl	5.218	/	/
713.		Pi-Alkyl	5.432	/	/
714.		Pi-Alkyl	5.247	[d] 43.953	[d] 48.107
715.		Pi-Alkyl	4.795	[d] 45.364	[d] 42.994
716.		Pi-Pi Stacked	4.053	[d] 38.288	[d] 30.063
717.		Pi-Pi Stacked	4.904	[d] 52.462	[d] 44.203
718.		Pi-Pi Stacked	5.405	[d] 53.506	[d] 49.469
719.		Pi-Pi Stacked	5.173	[d] 45.394	[d] 47.344
720.		Pi-Pi Stacked	3.802	[d] 26.440	[d] 22.687
721.		Pi-Pi Stacked	4.366	[d] 29.067	[d] 36.554
722.		Pi-Pi Stacked	5.060	[d] 37.715	[d] 46.238
723.		Pi-Pi Stacked	3.727	[d] 15.5456	[d] 19.721

No.	Mixture	Type of interaction	Distance (Å)	Angle1(º)	Angle2(º)
724.		Pi-Pi Stacked	5.239	/	/
725.		Pi-Alkyl	4.419	/	/
726.		Pi-Alkyl	4.364		
727.	py-GBC-TTC	Pi-Alkyl	4.873	/	/
728.		Pi-Alkyl	4.195	/	/
729.		Pi-Alkyl	4.883	/	/
730.		Pi-Alkyl	5.396	/	/
731.		Pi-Alkyl	4.886	[d]39.611	[d]51.452
732.		Pi-Pi Stacked	4.811	[d]44.245	[d]48.859
733.		Pi-Pi Stacked	4.275	[d]33.376	[d]42.482
734.		Pi-Pi Stacked	3.446	[d]15.349	[d]21.004
735.		Pi-Pi Stacked	4.998	[d]7.230	[d]50.159
736.		Pi-Pi Stacked	4.202	[d]42.914	[d]39.384
737.		Pi-Pi Stacked	3.573	[d]26.442	[d]25.108
738.		Pi-Pi Stacked	3.578	[d]32.626	[d]25.841
739.		Pi-Pi Stacked	4.963	[d]55.760	[d]49.295
740.		Pi-Pi Stacked	4.475	[d]49.208	[d]44.225
741.		Pi-Pi Stacked	3.010	[c]33.935	[c]3.010
742.		Pi-Donor Hydrogen Bond	2.649	[c]18.789	[c]2.649
743.		Pi-Donor Hydrogen Bond	3.373	[c]42.808	[c]3.373
744.		Pi-Donor Hydrogen Bond Pi-Sigma	2.911	[c]33.940	[c]2.911
745.		Pi-Sigma	2.773	[c]2.973	[c]2.773
746.		Pi-Pi Stacked	4.157	[d] 39.834	[d] 31.435
747.		Pi-Pi Stacked	3.727	[d] 24.178	[d] 17.884
748.		Pi-Pi Stacked	4.946	/	/
749.	gra-PBC-DMC	Pi-Alkyl	5.041	[d] 46.711	[d] 45.295
750.		Pi-Pi Stacked	3.866	[d] 18.640	[d] 23.450
751.		Pi-Pi Stacked	4.316	[d] 26.233	[d] 34.741
752.		Pi-Pi Stacked	4.004	/	/
753.		Pi-Alkyl	5.172	[d] 36.925	[d] 46.709
754.		Pi-Pi Stacked	5.489	[d] 44.338	[d] 49.741
755.		Pi-Pi Stacked	5.153	/	/
756.		Pi-Alkyl	4.520	/	/

No.	Mixture	Type of interaction	Distance (Å)	Angle1(º)	Angle2(º)
757.		Pi-Alkyl	2.888	/	/
758.		Pi-Lone Pair	4.444	d 41.644	d 38.027
759.		Pi-Pi Stacked	3.753	d 31.139	d 21.134
760.		Pi-Pi Stacked	3.965	d 26.587	d 27.997
761.	gra-PBC-DOX	Pi-Pi Stacked	3.787	d 15.134	d 22.433
762.		Pi-Pi Stacked	5.205	d 43.653	d 47.739
763.		Pi-Pi Stacked	4.864	/	/
764.		Pi-Alkyl	4.806	d 33.835	d 43.268
765.		Pi-Pi Stacked	4.809	/	/
766.		Pi-Alkyl	2.624	d 9.613	
767.		Pi-Sigma	4.508	/	/
768.		Pi-Alkyl	2.542	d 4.363	
769.		Pi-Sigma	4.257	/	/
770.		Pi-Alkyl	5.291	/	/
771.		Pi-Alkyl	5.169	d 42.454	d 46.659
772.	gra-PBC-CTC	Pi-Pi Stacked	5.422	d 37.109	d 49.121
773.		Pi-Pi Stacked	5.469	/	/
774.		Pi-Alkyl	4.947	d 30.850	d 44.188
775.		Pi-Pi Stacked	4.673	/	/
776.		Pi-Alkyl	3.834	d 12.853	d 22.299
777.		Pi-Pi Stacked	5.147	/	/
778.		Pi-Alkyl	4.368	d 42.358	d 35.665
779.		Pi-Pi Stacked	4.105	d 26.864	d 30.203
780.	gra-PBC-CTC	Pi-Pi Stacked	3.743	/	/
781.		Pi-Alkyl	3.760	d 33.001	d 19.347
782.		Pi-PI Stacked	5.071	/	/
783.		Pi-Alkyl	5.311	/	/
784.		Pi-Alkyl	3.809	/	/
785.		Pi-Alkyl	2.820	d 2.902	/
786.	gra-PBC-MC	Pi-Pi Stacked	4.192	/	/
787.		Pi-Alkyl	5.115	d 47.878	d 46.693
788.		Pi-Pi Stacked	3.307	/	/
789.		Pi-Alkyl	4.192	d 32.089	d 33.194

No.	Mixture	Type of interaction	Distance (Å)	Angle1(º)	Angle2(º)
790.		Pi-Pi Stacked	4.810	[d] 40.213	[d] 43.193
791.		Pi-Pi Stacked	4.591	/	/
792.		Pi-Alkyl	3.864	[d] 27.450	[d] 24.790
793.		Pi-Pi Stacked	3.658	[d] 13.991	[d] 16.452
794.		Pi-Pi Stacked	4.784	/	/
795.		Pi-Alkyl	4.542	/	/
796.		Pi-Alkyl	5.190	[d] 37.737	[d] 45.950
797.	gra-PBC-MN	Pi-Pi Stacked	4.659	/	/
798.		Pi-Alkyl	5.622	[d] 38.068	[d] 50.092
799.		Pi-Pi Stacked	4.790	/	/
800.		Pi-Alkyl	4.261	[d] 33.560	[d] 32.143
801.		Pi-Pi Stacked	4.113	/	/
802.		Pi-Alkyl	3.970	[d] 12.647	[d] 24.658
803.		Pi-Pi Stacked	5.004	/	/
804.		Pi-Alkyl	5.244	[d] 36.870	[d] 46.520
805.		Pi-Pi Stacked	5.128	/	/
806.		Pi-Alkyl	3.748	[d] 27.636	[d] 15.684
807.		Pi-Pi Stacked	5.228	/	/
808.		Pi-Alkyl	4.327	[d] 32.641	[d] 33.507
809.		Pi-Pi Stacked	4.499	[d] 48.295	[d] 38.210
810.		Pi-Pi Stacked	5.423	[d] 52.560	[d] 49.323
811.		Pi-Pi Stacked	3.685	[d] 27.695	[d] 16.386
812.	gra-PBC-OTC	Pi-Pi Stacked	3.961	[d] 25.795	[d] 26.808
813.		Pi-Pi Stacked	5.649	[d] 46.788	[d] 51.243
814.		Pi-Pi Stacked	4.012	[d] 17.008	[d] 28.220
815.		Pi-Pi Stacked	5.027	[d] 34.576	[d] 45.317
816.		Pi-Pi Stacked	2.532	[d] 4.982	/
817.		Pi-Sigma	5.326	/	/
818.		Pi-Alkyl	2.637	[d] 4.208	/
819.		Pi-Donor Hydrogen Bond	5.400	/	/
820.	gra-PBC-TG	Pi-Alkyl	5.121	/	/
821.		Pi-Alkyl	5.761	[d] 40.916	[d] 60.045
822.		Pi-Pi Stacked	4.999	/	/

No.	Mixture	Type of interaction	Distance (Å)	Angle1(º)	Angle2(º)
823.		Pi-Alkyl	4.244	/	/
824.		Pi-Alkyl	4.398	d 22.950	d 49.168
825.		Pi-Sigma	2.763	c26.785	/
826.		Pi-Donor Hydrogen Bond	2.817	c24.096	/
827.		Pi-Donor Hydrogen Bond	3.219	c35.755	/
828.	gra-PBC-TTC	Pi-Cation	4.759	c35.574	/
829.		Pi-Pi Stacked	3.753	d 32.102	d 21.754
830.		Pi-Pi Stacked	5.832	d40.912	d63.413
831.		Pi-Pi Stacked	5.422	d39.065	d60.600
832.		Pi-Pi Stacked	4.962	d43.513	d55.086
833.		Pi-Alkyl	3.858	d23.372	d43.815
834.		Pi-Alkyl	4.295	/	/
835.		Pi-Alkyl	4.322	/	/
836.		Pi-Alkyl	4.044	/	/
837.		Alkyl	4.754	/	/
838.		Alkyl	4.052	/	/
839.		Alkyl	5.368	/	/
840.		Pi-Alkyl	5.127	/	/
841.		Alkyl	4.967	/	/
842.	gra-GBC-DMC	Alkyl	4.422	/	/
843.		Alkyl	5.189	/	/
844.		Pi-Alkyl	4.852	/	/
845.		Pi-Sigma	3.541	c13.858	d18.444
846.		Pi-Alkyl	4.601	/	/
847.		Pi-Alkyl	4.556	/	/
848.		Pi-Alkyl	4.495	/	/
849.		Pi-Alkyl	4.741	/	/
850.		Pi-Alkyl	3.711	/	/
851.		Alkyl	5.487	/	/
852.		Pi-Alkyl	4.106	/	/
853.		Pi-Sigma	3.706	c 16.760	d28.638
854.	gra-GBC-DOX	Pi-Alkyl	4.714	/	/
855.		Pi-Alkyl	4.916	/	/

No.	Mixture	Type of interaction	Distance (Å)	Angle1(º)	Angle2(º)
856.	gra-GBC-CTC	Pi-Alkyl	4.767	/	/
857.		Alkyl	5.271	/	/
858.		Pi-Alkyl	5.159	/	/
859.		Pi-Alkyl	5.136	/	/
860.		Alkyl	3.673	/	/
861.		Alkyl	4.436	/	/
862.		Pi-Alkyl	4.026	/	/
863.	gra-GBC-MC	Pi-Sigma	3.435	[c] 9.626	[d] 9.407
864.		Pi-Alkyl	5.353	/	/
865.		Alkyl	3.976	/	/
866.		Pi-Alkyl	4.514	/	/
867.		Alkyl	5.480	/	/
868.		Alkyl	5.355	/	/
869.		Pi-Alkyl	5.076	/	/
870.		Pi-Alkyl	4.597	/	/
871.		Alkyl	4.991	/	/
872.	gra-GBC-MN	Alkyl	4.773	/	/
873.		Alkyl	4.530	/	/
874.		Pi-Alkyl	5.076	/	/
875.		Alkyl	5.042	/	/
876.		Alkyl	3.928	/	/
877.		Pi-Alkyl	4.166	/	/
878.		Alkyl	4.894	/	/
879.		Pi-Alkyl	3.869	/	/
880.		Pi-Alkyl	5.181	/	/
881.		Pi-Alkyl	5.443	/	/
882.		Pi-Alkyl	3.728	/	/
883.		Pi-Alkyl	4.874	/	/
884.		Pi-Alkyl	4.753	/	/
885.		Alkyl	5.338	/	/
886.	gra-GBC-OTC	Pi-Alkyl	4.706	/	/
887.		Alkyl	4.058	/	/
888.		Alkyl	4.833	/	/

No.	Mixture	Type of interaction	Distance (Å)	Angle1(º)	Angle2(º)
889.	gra-GBC-TG	Alkyl	4.763	/	/
890.		Alkyl	5.439	/	/
891.		Pi-Alkyl	4.634	/	/
892.		Pi-Sigma	3.670	[c] 10.814	[d] 11.616
893.		Alkyl	4.647	/	/
894.		Pi-Alkyl	5.384	/	/
895.		Alky	5.317	/	/
896.		Pi-Alkyl	4.354	/	/
897.		Pi-Alkyl	4.728	/	/
898.		Pi-Alkyl	4.689	/	/
899.		Pi-Alkyl	4.238	/	/
900.		Pi-Alkyl	4.993	/	/
901.		Pi-Alkyl	5.021	/	/
902.		Pi-Alkyl	3.910	/	/
903.	gra-GBC-TTC	Pi-Alkyl	4.165	/	/
904.		Pi-Alkyl	4.953	/	/
905.		Pi-Alkyl	4.605	/	/
906.		Pi-Alkyl	5.429	/	/
907.		Pi-Pi Stacked	5.903	[d]47.517	[d]58.976
908.		Pi-Pi Stacked	4.729	[d]39.273	[d]48.533
909.		Pi-Pi Stacked	4.169	[d]32.131	[d]40.099
910.		Pi-Pi Stacked	4.656	[d]45.226	[d]45.381
911.		Pi-Pi Stacked	3.381	[d]14.446	[d]18.294
912.		Pi-Pi Stacked	3.557	[d]26.878	[d]24.678
913.		Pi-Pi Stacked	4.135	[d]44.693	[d]37.293
914.		Pi-Pi Stacked	3.608	[d]35.214	[d]27.025
915.		Pi-Pi Stacked	4.506	[d]50.505	[d]44.442
916.		Pi-Pi Stacked	4.972	[d]8.253	[d]48.660
917.		Pi-Pi Stacked	2.963	[d]11.333	/
918.		Pi-Pi Stacked	3.051	[c]35.196	/
919.		Pi-Lone Pair	2.679	[c]21.016	/
920.		Pi-Donor Hydrogen Bond	3.051	[c]35.196	/

Note: a Angle DHA; b Angle HAY; c Angle Deviation; d Angle θ.

Table S7. The standard curves and recovery rates of tested TCs[a]

TCs	Standard curves[b]	R^2	Recovery rates
CTC	y=163235x-248119	0.9999	102%
DMC	y=292343x-387239	0.9997	97%
MC	y=359218x-404338	0.9999	99%
MN	y=483980x-730868	0.9990	99%
OTC	y=36487x+19674	0.9999	98%
TTC	y=44502x+28613	0.9999	101%
DOX	$y=681903x-10^6$	0.9990	98%
TG	$y=403193x-2\times10^6$	0.9990	99%

Note: [a] the presented data was obtained in the tested concentrations of 1~100 mg·L^{-1}; [b] x presents peak area of tested TCs in HPLC spectrogram, y denotes tested TCs concentration in the solution.

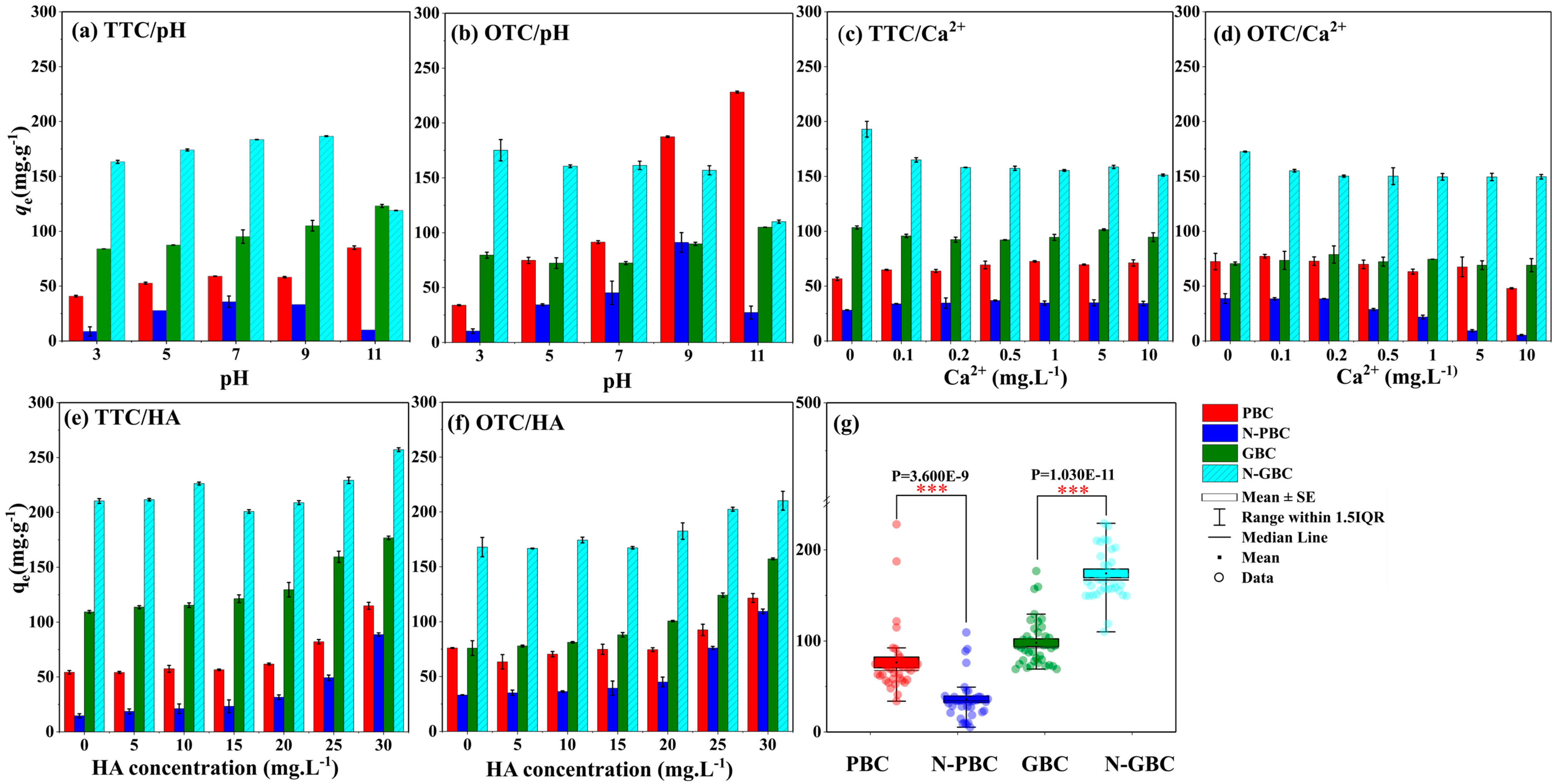

Figure S1. The influence of environmental factors (pH, Ca^{2+}, HA) on TTC and OTC adsorption efficiency (q_e) by tested biochars (a-f) and the statistical distribution of their adsorption efficiency (g).

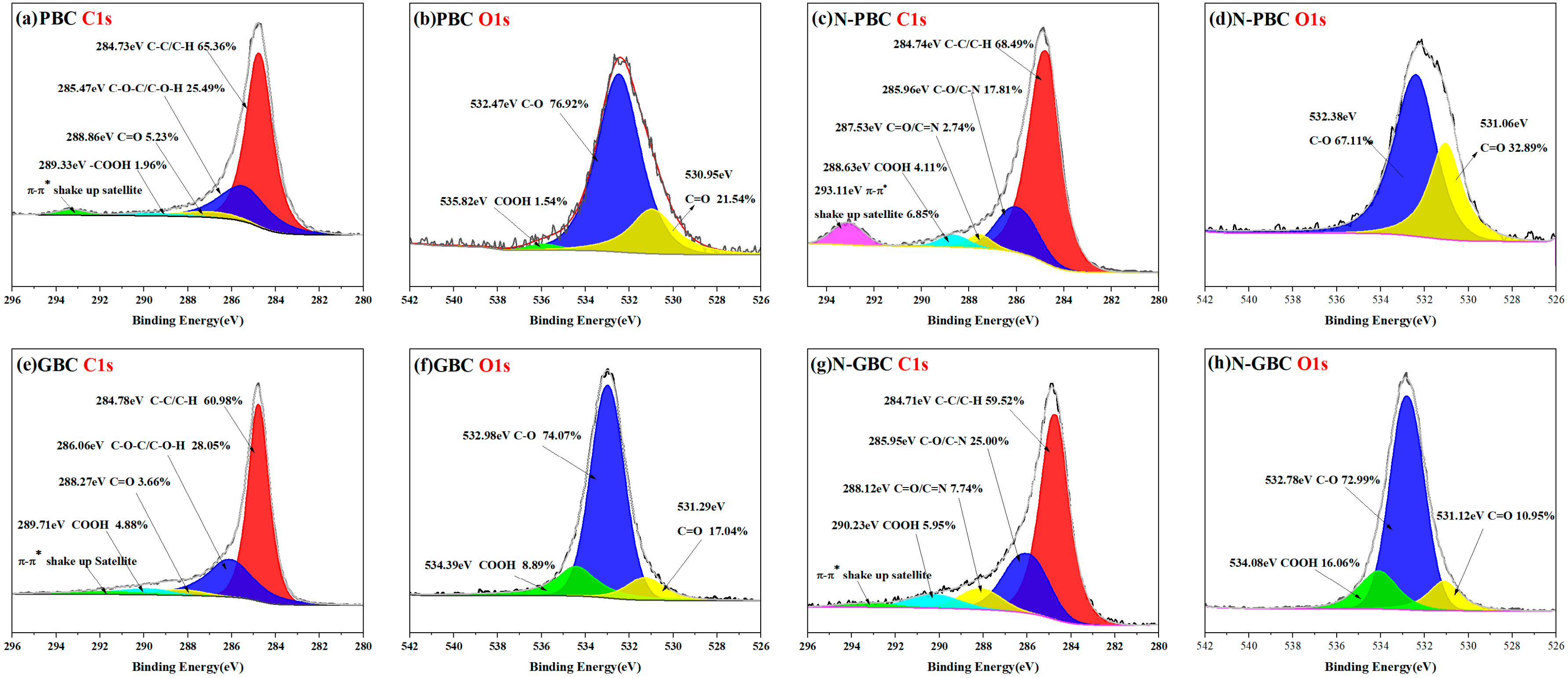

Figure S2. C1s, O1s peaks of tested biochars by XPS regional spectra.

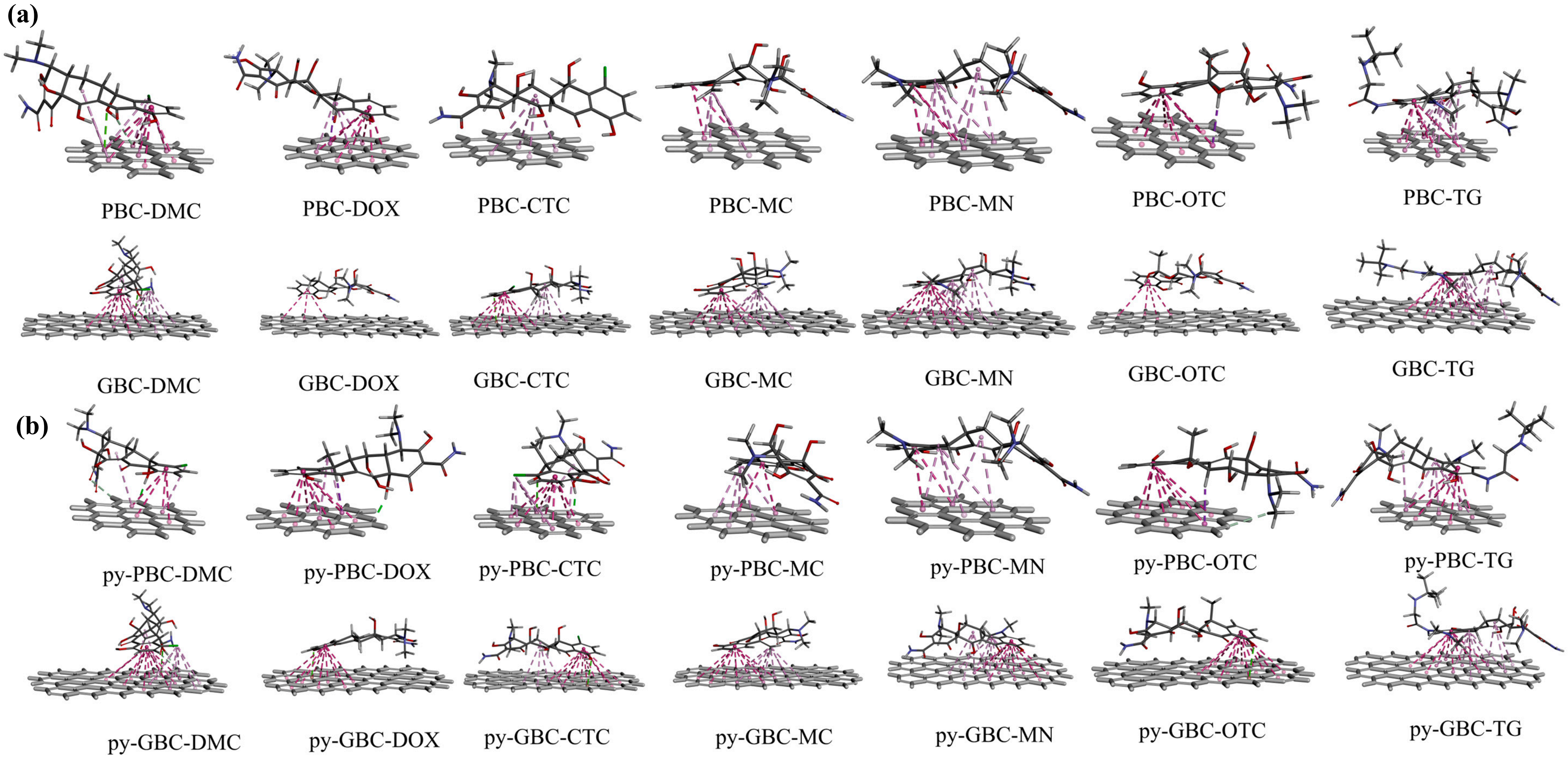
(a)
PBC-DMC
PBC-DOX
PBC-CTC
PBC-MC
PBC-MN
PBC-OTC
PBC-TG
GBC-DMC
GBC-DOX
GBC-CTC
GBC-MC
GBC-MN
GBC-OTC
GBC-TG
(b)
py-PBC-DMC
py-PBC-DOX
py-PBC-CTC
py-PBC-MC
py-PBC-MN
py-PBC-OTC
py-PBC-TG
py-GBC-DMC
py-GBC-DOX
py-GBC-CTC
py-GBC-MC
py-GBC-MN
py-GBC-OTC
py-GBC-TG

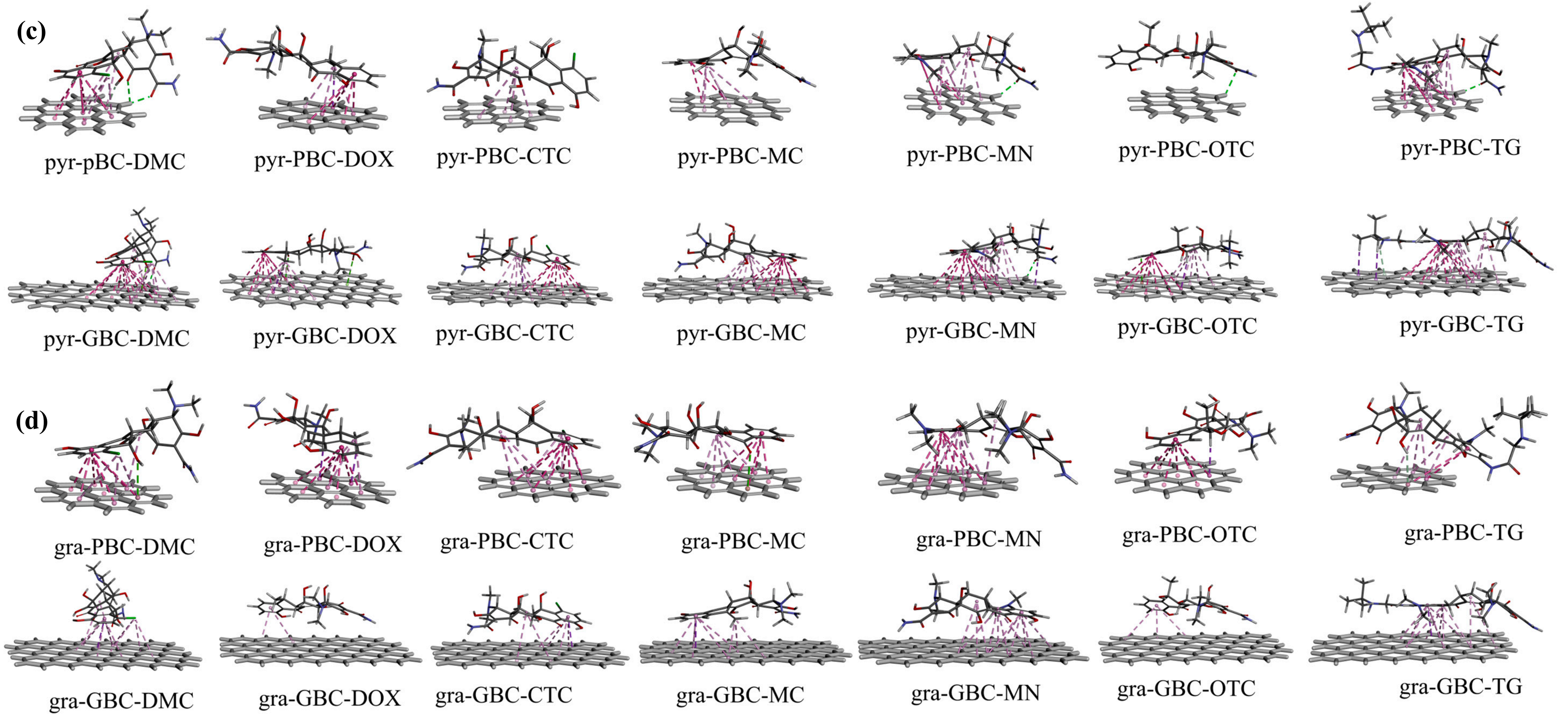

Figure S3. Equilibrium configurations of tested TCs (except TTC) adsorbed by PBC and GBC(a), adsorbed by py-PBC and py-GBC(b), adsorbed by pyr-PBC and pyr-GBC(c), adsorbed by gra-PBC and gra-GBC(d).

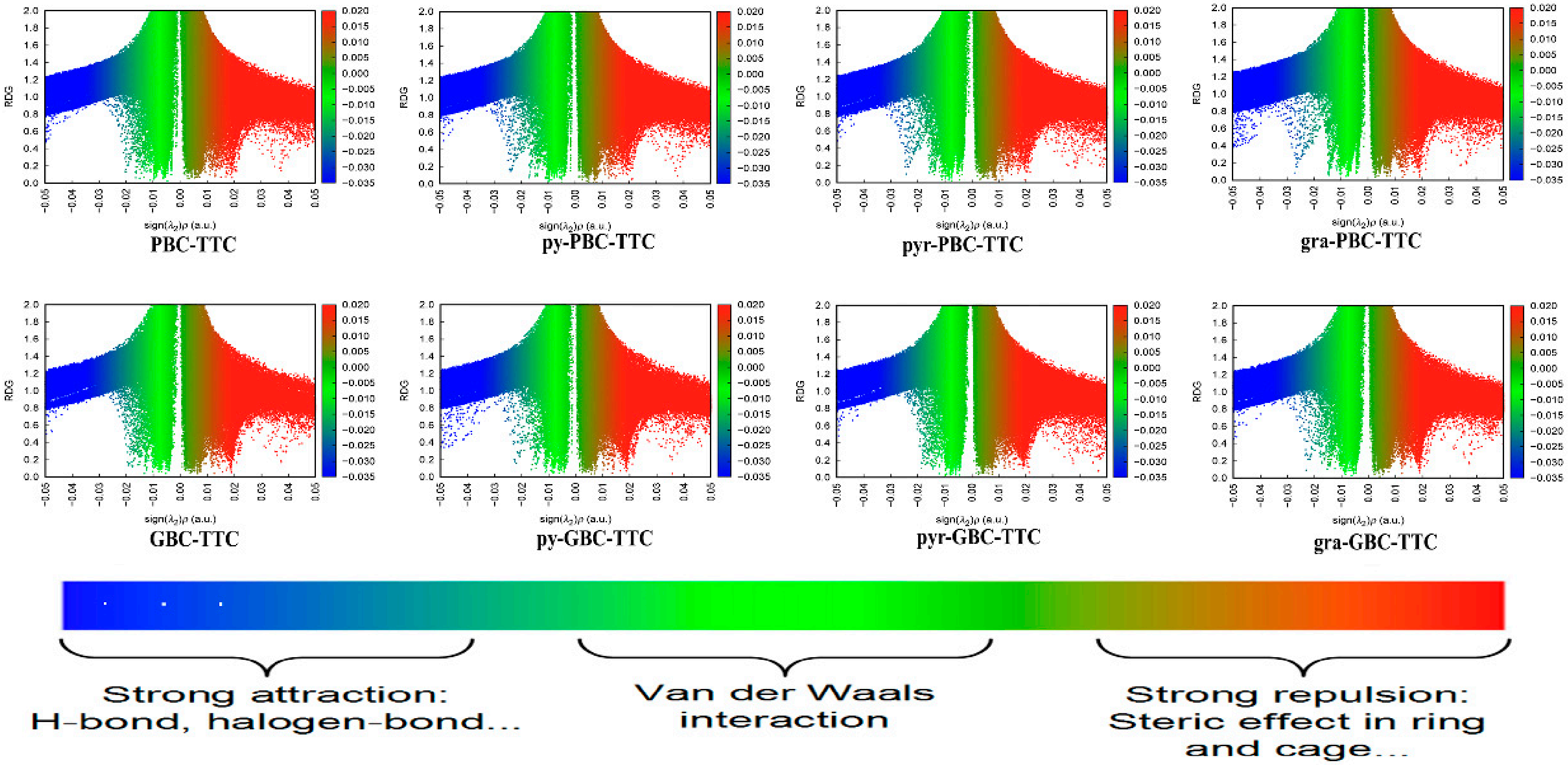

Figure S4 The scatterplots of reduced density gradients versus sign(λ2)*ρ* for the equilibrium configurations of TTC-biochars.

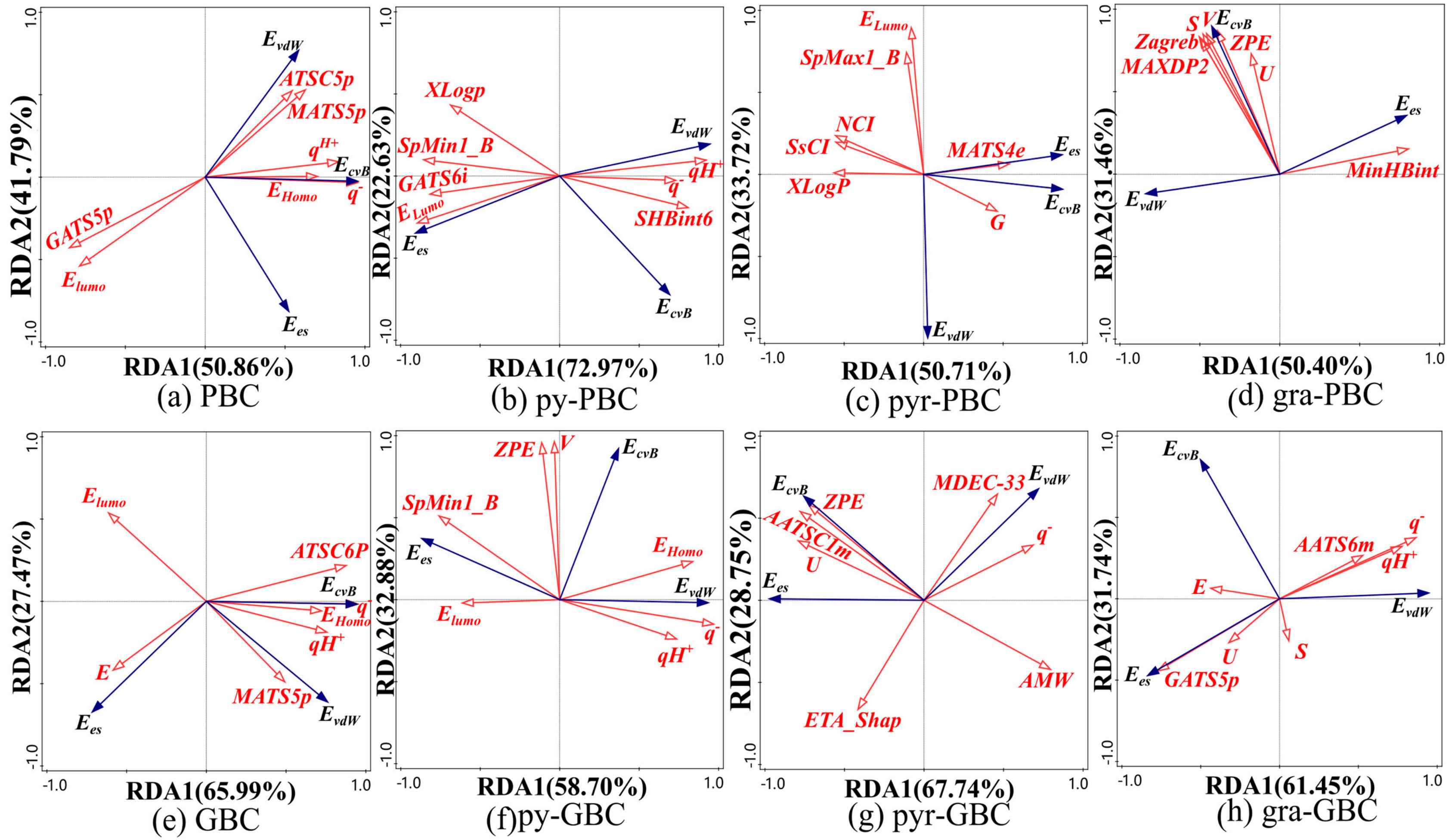

Figure S5. The redundancy analysis (RDA) of quantum chemical descriptors and E_{CVB}, E_{vdWs}, and E_{es}.

Reference

[1] T. Mahmood, M.T. Saddique, A. Naeem, P. Westerhoff, S. Mustafa, A. Alum, Comparison of different methods for the point of zero charge determination of NiO, Industrial & Engineering Chemistry Research 50(17) (2011) 10017-10023.

[2] Z. Liu, F. Feng, Y. Li, Y. Sun, K. Tagawa, A corncob biochar-based superhydrophobic photothermal coating with micro-nano-porous rough-structure for ice-phobic properties, Surface and Coatings Technology 457 (2023) 129299.

[3] Y.X. Fan, L.L. Huang, L.G. Wu, C.T. Zhang, S.H. Zhu, X.Y. Xiao, M. Li, X.M. Zou, Adsorption of sulfonamides on biochars derived from waste residues and its mechanism, J Hazard Mater.406 (2021) 124291.

[4] S. Manzetti, T. Lu, The geometry and electronic structure of Aristolochic acid: possible implications for a frozen resonance, J Phys Org Chem. 26(6) (2013) 473-483.

[5] T. Lu, F.W. Chen, Multiwfn: A multifunctional wavefunction analyzer, J Comput Chem. 33(5) (2012) 580-592.

[6] L. Lin, W.B. Jiang, P. Xu, Comparative study on pharmaceuticals adsorption in reclaimed water desalination concentrate using biochar: Impact of salts and organic matter, Sci Total Environ. 601 (2017) 857-864.
